# Performance of DNA methylation assays for detection of high-grade cervical intraepithelial neoplasia (CIN2+): a systematic review and meta-analysis

**DOI:** 10.1038/s41416-019-0593-4

**Published:** 2019-10-16

**Authors:** Helen Kelly, Yolanda Benavente, Miquel Angel Pavon, Silvia De Sanjose, Philippe Mayaud, Attila Tibor Lorincz

**Affiliations:** 1 Cancer Epidemiology Research Program, Catalan Institute of Oncology (ICO), IDIBELL, CIBER-ONC, L’Hospitalet de Llobregat, Barcelona, Spain; 2 London School of Hygiene and Tropical Medicine, London, UK; 3 CIBER en Epidemiología y Salud Pública (CIBERESP), Barcelona, Spain; 4 PATH, Seattle, WA USA; 5 Queen Mary University of London, Centre for Cancer Prevention, Wolfson Institute of Preventive Medicine, Barts & the London School of Medicine, London, UK

**Keywords:** Diagnostic markers, Cervical cancer

## Abstract

**Background:**

To conduct a meta-analysis of performance of DNA methylation in women with high-grade cervical intraepithelial neoplasia (CIN2+).

**Methods:**

Medline and Embase databases were searched for studies of methylation markers versus histological endpoints. Pooled sensitivity, specificity and positive predictive value (PPV) for CIN2+ were derived from bivariate models. Relative sensitivity and specificity for CIN2+ compared to cytology and HPV16/18 genotyping were pooled using random-effects models.

**Results:**

Sixteen thousand three hundred thirty-six women in 43 studies provided data on human genes (*CADM1, MAL, MIR-124-2, FAM19A4, POU4F3, EPB41L3, PAX1, SOX1)* and HPV16 (L1/L2). Most (81%) studies evaluated methylation assays following a high-risk (HR)-HPV-positive or abnormal cytology result. Pooled CIN2+ and CIN3+ prevalence was 36.7% and 21.5%. For a set specificity of 70%, methylation sensitivity for CIN2+ and CIN3+ were 68.6% (95% CI: 62.9–73.8) and 71.1% (95% CI: 65.7–76.0) and PPV were 53.4% (95% CI: 44.4–62.1) and 35.0% (95% CI: 28.9–41.6). Among HR-HPV+ women, the relative sensitivity of methylation for CIN2+ was 0.81 (95% CI: 0.63–1.04) and 1.22 (95% CI: 1.05–1.42) compared to cytology of atypical squamous cells of undetermined significance, or greater (ASCUS+) and HPV16/18 genotyping, respectively, while relative specificity was 1.25 (95% CI: 0.99–1.59) and 1.03 (95% CI: 0.94–1.13), respectively.

**Conclusion:**

DNA methylation is significantly higher in CIN2+ and CIN3+ compared to ≤CIN1. As triage test, DNA methylation has higher specificity than cytology ASCUS+ and higher sensitivity than HPV16/18 genotyping.

## Background

Invasive cervical cancer (ICC) is one of the most common female cancers in low and middle-income countries (LMIC), where 85% of the estimated 570,000 global annual cases occur and is the leading cause of cancer deaths among women in these settings.^1^ ICC is one of the most preventable and treatable forms of cancer, as long as it is detected early and managed effectively. In May 2018, the Director-General of the World Health Organization (WHO) made a global call for action towards the elimination of ICC calling for more innovative technologies for detection of precancerous lesions and better strategies to increase ICC screening coverage and uptake.^2^ There is strong evidence that high-risk human papillomavirus (HR-HPV) DNA based screening is more sensitive for the detection of high-grade CIN (CIN2+) and is effective in prevention of ICC compared to cervical cytology and visual inspection.^3^ However, HPV testing detects many transient infections, meaning that its specificity for high-grade CIN is low,^4^ which has important implications for screening women with high prevalence of HR-HPV. Novel methods are required that are sensitive enough to detect clinically relevant HPV needing colposcopy referral but with high specificity to rule out HPV-positive women without evidence of disease, thereby avoiding repeat testing which can result in substantial loss to follow-up,^5^ as well as avoiding unnecessary referrals for colposcopy, which increase the workload and costs to the services. DNA methylation of human genes and HPV virus occur during HR-HPV infection and precancerous tissue progression, leading to alterations in the functions of gene products regulating tumour suppression.^6,7^ Such aberrant DNA methylation may help distinguish non-progressive HPV infections from those that will progress to cancer. Increased DNA methylation has been shown to be associated with increasing persistence of HR-HPV genotypes,^8^ severity of CIN lesions^9^ and risk of invasive cancer.^10^

Recent studies evaluating DNA methylation of human genes and the HPV virus for detection of HPV related lesions included different human genes and HPV genotypes. Furthermore, these studies varied in the CpG (cytosine followed by guanine) dinucleotide sites chosen, many of which occur in the human genome, in contrast to the HPV genome, which does not have any clearly discernible CpG islands.^6^ Previous systematic reviews have summarised the association and performance of DNA methylation for CIN2+ and CIN3+ detection,^6,8,9,11^ although none have yet quantified the performance in a meta-analysis.

The aim of this review and meta-analysis is to evaluate the performance of various DNA methylation markers (human genes and HPV virus) for detection of CIN2+ and CIN3+. The novelty of our review is that it evaluates: (i) the association of host and HPV methylation positivity with increasing grades of CIN (*Analysis 1*); (ii) the pooled sensitivity, specificity and positive predictive value (PPV) of DNA methylation markers for the detection of CIN2+ and CIN3+ in a triage setting (i.e. following HR-HPV-positive test or abnormal cytology; *Analysis 2*) and (iii) the relative sensitivity and relative specificity of DNA methylation markers compared to HPV16/18 genotyping and cytology (using a cut-off of atypical squamous cells of undetermined significance, or greater [ASCUS+] or low-grade squamous intraepithelial lesions, or greater [LSIL+]), for the detection of CIN2+ and CIN3+ in a triage setting (*Analysis 3*).

## Methods

### Study outcomes

Studies were included if they reported the percentage of DNA methylation according to CIN grade, or sensitivity and specificity of the DNA methylation assays for the detection of the outcome, or if the receiver operating characteristic (ROC) curve was provided from which sensitivity and specificity estimates could be obtained.

Studies were included if methylation markers were assessed against a histological endpoint of CIN grade 2 or higher (CIN2/3, CIN2+ or CIN3+ which can include carcinoma in situ and ICC). Studies with cytological endpoint assessment only were excluded because of the lower sensitivity for cytology measures in detection of high-grade disease.^12^

### Search

Medline, Embase and Cochrane databases were searched using the following search terms: DNA methylation [Title/Abstract] OR epigenetic [Title/Abstract] OR methylation [MeSH Terms] AND nucleic acids [MeSH Terms] OR CpG islands [MeSH Terms] AND HPV [Title/Abstract] OR human papillomavirus [Title/Abstract] OR carcinogenesis [MeSH Terms] OR Cervical Intraepithelial Neoplasia/ or intraepithelial neoplasia [Title/Abstract]. The search included all papers published up to 10 December 2018. All abstracts were screened by one author (HK). Full-text copies of relevant publications were obtained and assessed for eligibility by two authors (H.K. and A.L.). Consensus was reached on potential relevance.

### Inclusion and exclusion criteria

Studies reporting methylation within biopsy specimens were excluded as we aimed to evaluate the performance of DNA methylation assays as a potential primary screening or triage tests when cervical swabs would be used. Studies that reported only crude percentage methylation estimates without a validated cut-off for CIN2+ detection were excluded as they were not verified or validated. Studies were excluded if cancers represented greater than 10% of all samples and it was not possible to separately analyse the cancers, the CIN2 and the CIN3, due to the risk of spectrum bias related to the fact that the majority of cancers have very high levels of methylation.

Studies not in the English language or conference abstracts were excluded due to difficulty in assessing the quality of the methodology, as were studies with fewer than 25 participants, which could result in an unacceptably imprecise effect measurement. Whereby publications provided DNA methylation measures using a combination set of gene markers, the DNA methylation of the individual markers as well as the combination panel were presented separately in the results. The combination tests were positive when any of the included gene markers were positive.

Our review was restricted to DNA methylation markers where there were 4 or more studies evaluating the performance of an individual marker (to reduce the potential heterogeneity when pooling a small number of studies), or if the marker had been evaluated as part of a large population-based screening study. Studies reporting only DNA methylation of HPV16 were included given the small number of studies evaluating DNA methylation of other HPV types.

### Data extraction

From the consensus list, data were extracted by one author (HK) using a standardised form. For all studies, the following variables were recorded: year of study, study location, origin (country) of study population, outcomes of interest (histological confirmed lesion CIN2+/CIN3+/ICC), DNA methylation marker evaluated, DNA methylation positive among CIN2+/CIN3+ (true positives), and ≤CIN1 (false-positives), and DNA methylation negative among ≤CIN1 (true-negatives) and CIN2+/CIN3+ (false-negatives), where given.

### Statistical analysis

#### Analysis 1

The percentage methylation (methylation positivity) was extracted for each grade of CIN (≤CIN1, CIN2, CIN3 and ICC) according to pre-defined thresholds established or if pre-defined thresholds were not available, methylation positivity was calculated from ROC curves based on a set specificity of 70% for CIN2+/CIN3+ detection by one author (HK) and validated by a second author (AL). Crude (unadjusted) Odds Ratios (OR) and 95% Confidence Intervals (CI) were calculated for methylation positivity associated with each grade of discrete grades of high-grade CIN (CIN2, CIN3 and ICC) compared to CIN1 or normal (≤CIN1). Random-effects meta-analysis were used to estimate pooled effects to account for between-study heterogeneity and heterogeneity was examined using the *I*^2^ statistic.^13^ Sub-group analyses by DNA methylation marker were performed to compare pooled effects and heterogeneity.

#### Analysis 2

The numbers of true positives, false positives, true negatives and false negatives were extracted where available, obtained using study-specific thresholds to define methylation positivity. Where several thresholds for methylation positivity were reported or where only ROC curves were presented, sensitivity data were extracted based on a threshold that produced a pre-defined set specificity of 70% and separately a set specificity of 50%.

The bivariate model^14^ was used to estimate pooled sensitivity and specificity using metandi and midas in STATA, whereby pairs of sensitivity and specificity are jointly analysed, incorporating any correlation that might exist between these two measures using a random-effects approach. Individual meta-analyses were performed for each of the human gene and HPV methylation markers. Because methylation markers are not independent of each other and given that most methylation markers perform better combined in a panel, a meta-analysis of combination markers was also performed, where available. To account for correlation between sensitivity and specificity, we used the hierarchical summary receiver operating characteristic (HSROC),^15^ which allows for threshold effects and between- and within-study variability, by allowing both test accuracy to vary across studies. Heterogeneity in the Forest Plots was assessed by visually examining the confidence intervals of individual studies.

A bivariate logitnormal random-effects model^16^ was used to estimate pooled PPV from the observed prevalence of CIN2+ and CIN3+ (Model 1). To account for varying observed CIN2+/CIN3+ prevalence in included studies, the pooled sensitivity and specificity estimates obtained using the bivariate model^14^ were used to generate a pooled PPV for varying expected CIN2+ and CIN3+ prevalence using PPV = Prev*SE/[Prev*SE+(1 − Prev)*(1 − Spec)]^17^ (Model 2). We assumed no change in performance of DNA methylation assays with increasing prevalence of CIN2+/CIN3+.

#### Analysis 3

Relative sensitivity and relative specificity and 95% Confidence Interval (CI) of DNA methylation assays for CIN2+ and CIN3+ detection were compared to other test strategies most widely reported, including HPV16/18 genotyping and cervical cytology (ASCUS+ and LSIL+) evaluated as triage tests following a HR-HPV-positive test. Where studies did not restrict inclusion to HR-HPV-positive women only, the performance of DNA methylation assays was compared to that of qualitative HR-HPV DNA-based tests (Hybrid Capture II or PCR). Only those studies that provided direct head-to-head comparison of the two methods on the same population were included. The data on true positive, false positive, true negative and false negative for each test method and for each study were extracted into Excel spreadsheet and imported into SAS. The sensitivity and specificity of DNA methylation were compared to that cytology and/or HPV16/18 genotyping using metadas in SAS,^18^ which allows comparison of test method through inclusion of test method as a covariate.^19^ We used sensitivity estimates for DNA methylation assays based on a threshold to achieve 70% specificity where studies allowed.

For each of the three analyses, separate sub-analyses were conducted for discrete outcomes of CIN2+ and CIN3+. Data were analysed using Stata (version 16) and SAS (version 9.4).

### Methodological quality assessment

Study quality was assessed using the QUADAS-2 tool for the quality assessment of diagnostic accuracy studies.^20^ Assessments were based on: participant selection characteristics (location, inclusion and exclusion criteria, study size and age distribution); proportion of women with CIN2+/CIN3+ included; whether the index test (DNA methylation assay) and reference test (histology) were well described; indication for biopsy (i.e. whether all women had biopsy taken irrespective of screening or triage test abnormality) and whether there was independent validation of histopathology diagnosis (Supplementary Tables 1, 2).

Studies were ranked in quality/robustness of design (linked to evidence for effectiveness of cervical cancer screening) in decreasing order of randomised clinical trial or randomised population-based trial, cohort studies, case-control studies and convenience sampling studies.^21^

Our review was reported according to the Preferred Reporting Items for Systematic Reviews and Meta-Analyses (PRISMA)^22^ and the Meta-analysis of Observational Studies in Epidemiology (MOOSE) guidelines.^23^ This review is registered on the PROSPERO database at the Centre of Reviews and Dissemination, University of York; registration number CRD42016052119. The full dataset is available online at (10.17632/84khm3rf8k.1).

## Results

### Characteristics of included studies

**Fig. 1:**
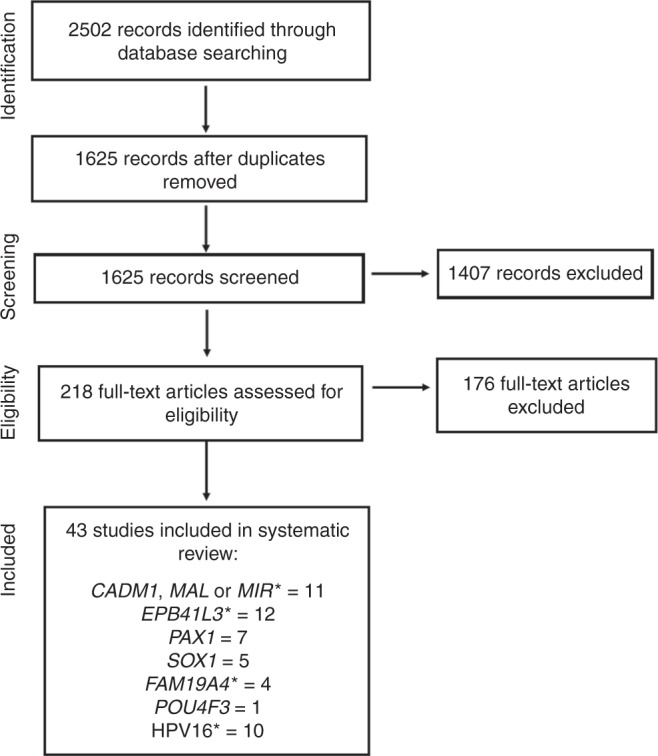
Flowchart for study selection. *studies evaluated a combination of human genes and/or HPV16.

The review identified 2502 publications through Medline, Embase and Cochrane library searches, which reported the association of the methylation of human genes or of any HPV type with any of the following outcome groups: CIN2/3, CIN3, CIN2+ and CIN3+ (including ICC) (Fig. 1), of which 877 were duplicates and 1407 were excluded after abstract review, leaving 218 articles for full-text review. Finally, 43 articles were selected which matched the inclusion criteria, among which there were at minimum four reports for any single gene, with the exception of one study evaluating *POU4F3* in a large population-based screening study.^24^ One study included discrete populations in two countries^25^ and is considered as two separate studies. The characteristics of these studies are summarised in Supplementary Table 2.

Among the 43 included studies and 16,336 women, nine (20.9%) evaluated DNA methylation as part of population-based screening among women with screening test abnormality (from here on referred to as a referral-population-based  studies; eight among HR-HPV-positive women^24,26–32^ and one among HPV16-positive women with abnormal cytology^33^), eight (18.6%) among women enrolled in cross-sectional or prospective cohort studies,^34–41^ six (14.0%) using a case-control design,^10,25,42–44^ eleven (26%) using convenience sampling^30,45–54^ and nine (21%) among women with HPV16 infection (two cohort,^55,56^ four case-control^57–60^ and three convenience studies;^61–63^ Table 1). There was one randomised controlled non-inferiority trial^64^ not included in the review, which randomly allocated women who tested positive for HR-HPV to either triage by cytology or DNA methylation. As the threshold was pre-defined and ROC curve was not provided, an earlier study by the same authors among the same cohort and which provided ROC was included in the analysis.^27^

**Table 1:** Characteristics of 43 included studies

Author, year	Study ID	Country	Study design	First screen test	Sample	Median age (IQR/range)	n CIN2+ (%)^a^	n ICC (%)	Genes studied	Markers evaluated singly/in combination	Threshold for methylation positivity pre-defined
Van Zummern 2017^34^	C2	South Africa-Pretoria	Cohort	None	321	40 [IQR; 35–46]	94 (29.3%)	2 (0.6%)	*CADM1/MAL/miR124-2*	Combination only	Yes
Kim 2016^45^	C3	South Korea-Seoul	Convenience	HPV+	122	NR	48 (39.3%)	0 (0.0%)^b^	*ADM1, MAL, PAX1*	Single only	Yes
Van Baars 2016^46^	C4	Spain-Barcelona	Convenience	Abnormal cytology	60	33 [19–92]	25 (41.7%)	0 (0.0%)	*CADM1/MAL*	Combination + single	Yes
De Vuyst 2015^35^	C5	Kenya-Nairobi	Cohort	HR-HPV (Genotyping)	248	37 [33–42]	93 (37.5%)	0 (0.0%)	*CADM1/MAL/miR124-2*	Combination + single	Obtained from ROC
Verhoef 2015^26^	C6	The Netherlands	Population-based screening—referral population	HR-HPV	364	42 [38–48]	90 (24.7%)	6 (1.6%)	*CADM1/MAL*	Combination only	Yes
De Strooper 2014^47^	C7	The Netherlands	Convenience	None (HR-HPV >80%)	168	37–48	48 (28.6%)	0 (0.0%)^b^	*CADM1/MAL/MIR124-2*	Combination only	Yes
De Strooper 2014^48^	C8	The Netherlands	Convenience	HR-HPV	234	34–40	58 (24.8%)	4 (1.7%)	*CADM1/MAL*	Combination only	Obtained from ROC
Verhoef 2014^27^	C9	The Netherlands	Population-based screeningreferral population	HR-HPV	1019	43 [33–63]	225 (22.1%)	13 (1.3%)	*MAL/MIR-124-2*	Combination only	Yes
Hesselink 2014^32^	C10	The Netherlands	Population-based screening– referral population	HR-HPV	355	41 [31–62]	94 (26.5%)	5 (1.4)	*CADM1/MAL/MIR124-2*	Combination + single	Obtained from ROC
Hesselink 2011^49^	C11	The Netherlands	Convenience	HR-HPV	236	40 [19–62]	58 (24.6%)	4 (1.7)	*CADM1/MAL*	Combination + single	Yes
Overmeer 2011^50^	C12	The Netherlands	Convenience	HR-HPV	70	34 [21–52]	30 (42.9%)	2 (2.9)	*CADM1/MAL*	Combination + single	Yes
De Strooper 2016^30^	F1	The Netherlands	Population-based screening – referral population	HR-HPV	254	36 (30–62)	99 (39.0%)	4 (1.6)	*FAM194A/MIR1242*	Combination only	Yes
Luttmer 2016^31^	F2	The Netherlands	Population-based screening– referral population	HR-HPV	508	~30yrs	180 (35.4%)	3 (0.6)	*FAM194A*	Single only	Yes
De Strooper 2014^65^	F3	The Netherlands	Convenience	HR-HPV	218	39 (19–62)	52 (23.9)	3 (1.4)	FAM194A	Single only	Yes
Bu, 2018^c^ ^37^	F4	China-Guangdong	Cohort	HR-HPV	154	NR	57 (37.0)	0 (0.0)	FAM194A	Single only	Obtained from ROC
Kocsis 2017^24^	P1	Hungary	Population-based screening– referral population	HR-HPV	1287	36 (25–40)	91 (7.1%)	12 (0.9%)	*POU4F3*	Single only	Yes
Kelly 2018 (BF)^25^	E1	Burkina Faso-Ouagadougou	Case-control	None (HR-HPV >80%)	94	39 (35–43)	28 (29.8)	1 (1.0%)	*EPB41L3*	Single only	Obtained from ROC
Kelly 2018 (SA)^25^	E1	South Africa- Johannesburg	Case-control	None (HR-HPV >80%)	268	33 (30–38)	124 (46.3)	0 (0.0%)	*EPB41L3*	Single only	Obtained from ROC
Lorincz 2016^42^	E2	UK-London	Case-control	HR-HPV (Aptima)	341	NR	39 (11.4)	1 (0.3%)	*EPB41L3/*HPV-16/-18/-31/-33 (S5 classifier)	Combination only	Pre-defined + ROC
Louvanto 2015^57^	E3	Canada-Montreal	Case-control	HPV16 positive	151	NR	94 (62.3)	0 (0.0%)^b^	*EPB41L3*, HPV16	Single only	Yes
Vasiljevic 2014^28^	E4	UK-London	Population-based screening– referral population	HR-HPV	1429	NR	531 (37.2)	0 (0.0%)	*EPB41L3*	Single only	Obtained from ROC
Boers 2014^51^	E5	The Netherlands	Convenience	HR-HPV	128	NR	34 (26.6)	9 (7.0)	*EPB41L3*	Single only	Pre-defined + ROC
Eijinsk 2012^52^	E6	The Netherlands-Groningen	Convenience	HR-HPV	200	20–85	66 (33.0)	0 (0.0%)^b^	*EPB41L3*	Single only	Yes
van Leeuwan 2018^29^	E7	Slovenia	Population-based screening– referral population	HR-HPV	235	NR	35 (14.9)	3 (1.3)	*EPB41L3, SOX1*	Single	Yes
Cook 2018^10^	E8	Canada-British Colombia	Case-control	HR-HPV	257	NR	107 (41.6)	0 (0.0)	*EPB41L3/*HPV-16/-18/-31/-33 (S5 classifier)	Combination	Obtained from ROC
Boers 2016^53^	E9	The Netherlands-Groningen	Convenience	Cytology ASCUS+	171	20–85	106 (62.0)	0 (0.0%)^b^	*EPB41L3*	Single	Yes
Rogeri 2018^36^	E11	Brazil-Sao Paulo	Cohort	None (63% HR-HPV-positive)	402	NR	126 (31.3)	0 (0.0)	*EPB41L3, SOX1*	Single only	Yes
Xu 2015^43^	PS1	China-Shanghai	Case-control	None (HR-HPV >76%)	94	36–44	34 (36.2)	0 (0.0%)^b^	*PAX1*	Single only	Obtained from ROC
Kan 2014^38^	PS2	Taiwan-Taipei	Cohort	None	419	NR	43 (10.3)	4 (1.0)	*PAX1, SOX1*	Single only	Yes
Lai 2014^44^	PS3	Taiwan	Case-control	Abnormal cytology	346	46	92 (26.6)	30 (8.7)	*PAX1, SOX1*	Single only	Pre-defined + ROC
Li 2015^39^	PS4	China-Weifang	Cohort	Abnormal cytology (ASCUS+)	463	46 (25–68)	34 (7.3)	2 (0.4%)	*PAX1*	Single only	Pre-defined + ROC
Lin 2011^40^	PS5	Taiwan	Cohort	Abnormal cytology (ASCUS+)	220	NR	42 (19.1)	11 (5.0)	*PAX1*	Single only	Yes-but unclear how
Huang 2010^54^	PS6	Taiwan	Convenience	Abnormal cytology	59		32 (54.2)	0 (0.0)	*PAX1*	Single only	Yes
Tian 2017^41^	PS8	Taiwan	Cohort	HR-HPV	173	NR	79 (45.7)	17 (9.8)	*PAX1, SOX1*	Combination	Yes
Bryant 2015^58^	H16-1	UK-Cardiff	Case-control (HPV16+)	HPV16 positive	200	21 (mean)	145 (72.5)	2 (1.0%)	HPV16-L1/L2	Single only	Obtained from ROC
Mirabello 2015^59^	H16-2	USA-California	Case-control (HPV16+)	HPV16 positive	99	34 [21–64]	59 (59.6)	0 (0.0%)	HPV16-L1	Single only	Obtained from ROC
Qiu 2015^61^	H16-3	China-Zhengzhou	Convenience (HPV16+)	HPV16 positive	114	37 [25–74]	72 (63.2)	11 (9.6)	HPV16-L1	Single only	Yes
Simanaviciene 2015^62^	H16-4	Lithuania-Vilnius	Convenience (HPV16+)	HPV16 positive	126	NR	87 (69.0)	0 (0.0%)^b^	HPV16-L1	Single only	Yes
Brandsma 2014^63^	H16-5	Senegal-Dakar/USA-New Haven	Convenience (HPV16+)	HPV16 positive	33	34 (23–65)	12 (36.4)	0 (0.0%)^b^	HPV16-L1/L2/E2	Single only	Yes
Brentnall 2014^33^	H16-6	UK-London	Population-based screening– referral population	Abnormal cytology and HPV16+	1493	NR	556 (37.2)	0 (0.0%)	*EPB41L3/*HPV-16/-18/-31/-33 (S5 classifier)	Combination only	Obtained from ROC
Lorincz 2013^55^	H16-7	UK-Wales	Cohort (HPV16+)	HPV16 positive	73	NR	25 (34.2)	0 (0.0%)	HPV16-L1/L2	Single only	Pre-defined + ROC
Mirabello 2013^60^	H16-8	Costa-Rica-Guanacaste	Case-control (HPV16+)	Abnormal VI or cyto and HPV16+	273	NR	109 (39.9)	13 (4.8%)	HPV16 L1	Single only	Obtained from ROC
Kottaridi 2017^56^	H16-9	Greece-Athens & UK-London	Case-control (HPV16+)	HPV16 positive	150	36 (21–62)	115 (76.6)	9 (7.8)	HPV16 L1	Single only	Yes

*ROC* Receiver Operating Characteristics; *ASCUS*+ atypical squamous cells of undetermined significance, or greater; *VI* visual inspection; *IQR* interquartile range

^a^CIN2+ prevalence among women with a HR-HPV DNA positive test or cytology abnormality or among studies with high prevalence HR-HPV

^b^Cancers were excluded from the analyses due to the high proportion (>10% of all samples)

^c^For Bu et al 2018 (F4), 61 (28% of all samples) were cervical cancer cases and were excluded to reduce bias ; Brentnall et al, 2014 (HPV16-6) is evaluated for HPV16 DNA methylation (restricted to HPV16-positive women)

Overall, 20 (47%) studies evaluated DNA methylation markers among women with a HR-HPV DNA-positive result,^10,24,26–32,35,37,41,42,45,48–52,65^ seven (16%) among women with an abnormal cytology^33,39,40,44,46,53,54^ and nine (21%) among women with HPV16 infection^55–63^ (Table 1). Of the remaining seven studies, five evaluated DNA methylation as primary screening in cohort or case-control studies with high prevalence of HR-HPV (42%,^34^ 63%,^36^ 76%^43^ and 80%^25^) and one in a convenience study (80% HR-HPV^47^). One study did not provide any data on HR-HPV or cytology outcomes.^38^

The pooled (unadjusted) CIN2+ positivity among 12,552 women in 38 studies was 36.7% and was higher in convenience studies and in studies involving HPV16-positive women (27.2%, 24.3%, 33.0%, 36.4% and 58.3% in referral-population-based, cohort, case-control, convenience and HPV16-positive women studies, respectively; Table 2). The pooled (unadjusted) CIN3+ positivity among 7393 women in 30 studies was 21.5% and was also higher in convenience and HPV16-positive women studies (17.4%, 17.7%, 14.8%, 21.5% and 43.1% in referral-population-based, cohort, case-control, convenience and HPV16-positive women studies, respectively).

**Table 2:** Meta-analysis of the performance of DNA methylation assays for detection of CIN2+ and CIN3+

	*N* studies	*N* women	Pooled CIN2+/CIN3+ prevalence	Pooled sensitivity, % (95% CI)	*I2*	Pooled specificity, % (95% CI)	*I2*
*CIN2+ detection*							
All studies, irrespective of threshold level^10,24–31,33–40,42,43,45–49,52–63,65^ ^a,b^	38	12,552	36.7%	63.2 (56.4–69.5)	*90.5* (*88.2–92.8)*	75.9 (71.9–79.5)	*85.7* (*81.9–89.6)*
Population-based screening studies	8	6589	27.2%	66.5 (56.8–75.0)	*93.0* (*89.6–96.5)*	70.8 (69.4–72.2)	*42.9* (*0.0–89.5)*
Cohort studies	7	2227	24.3%	68.0 (57.0–77.3)	*83.0* (*71.5–94.6)*	76.7 (66.3–84.6)	*95.3* (*93.1–97.5)*
Case-control studies^c^	5	1054	33.0%	58.7 (48.1–68.5)	*34.2* (*0.0–98.4)*	78.2 (62.8–88.4)	*76.5* (*55.7–97.4)*
Convenience studies^c^	9	1468	36.4%	45.9 (31.7–60.9)	*87.2* (*79.7–94.7)*	84.1 (74.8–90.4)	*83.5* (*73.0–93.9)*
HPV16-positive samples^d^	9	1214	58.3%	71.8 (54.5–84.4)	*95.3* (*93.3–97.4)*	73.5 (66.6–79.4)	*57.1* (*23.3–90.9)*
Set threshold to achieve 70% specificity^10,24–29,31,33,35,37,39,42,46,48,55–60,63,65^ ^e^	24	9646	35.9%	68.6 (62.9–73.8)	*86.3* (*81.7–90.9)*	70.5 (69.3–71.6)	*0.0* (*0.0–59.1)*
Set threshold to achieve 50% specificity^10,25,27,28,33–35,37,39,42,48,49,55,57,58,60^ ^e^	17	7225	34.9%	80.3 (75.6–84.4)	*83.5* (*76.2–90.7)*	50.1 (48.7–51.5)	*0.0* (*0.0–74.0)*
*CIN3+ detection*							
All studies, irrespective of threshold level^10,25–27,29–32,34,35,38,40–42,44,46–54,56,58,62,63,65^ ^a, f^	30	7393	21.5%	70.5 (64.8–75.6)	*80.0* (*73.3–86.6)*	74.7 (70.8–78.1)	*86.2* (*82.1–90.4)*
Referral-population-based studies	6	2708	17.4%	67.6 (60.4–74.0)	*67.2* (*38.8–95.6)*	70.1 (67.8–72.3)	*38.2* (*0.0–95.2)*
Cohort studies	5	1331	17.7%	78.2 (68.3–85.7)	*77.7* (*58.2–97.3)*	76.4 (61.9–86.6)	*97.0* (*95.5–98.5)*
Case-control studies	5	1306	14.8%	71.0 (60.0–80.0)	*61.6* (*24.0–99.2)*	70.0 (67.0–73.0)	*0.0* (*0.0–100.0)*
Convenience studies	10	1544	21.5%	72.2 (64.5–78.8)	*54.0* (*21.1–86.8)*	76.6 (71.1–81.2)	*73.1* (*56.0–90.2)*
HPV16-positive samples	4	504	43.1%	58.8 (34.7–79.3)	*92.6* (*86.9–98.2)*	80.0 (59.4–91.6)	*83.5* (*68.2–98.9)*
Set threshold to achieve 70% specificity^10,25–27,29,31,32,35,41,42,44,46,48,51,53,58,63,65^ ^e^	19	5197	19.8%	71.1 (65.7–76.0)	*64.0* (*45.9–82.1)*	69.8 (68.3–71.3)	*0.0* (*0.0–69.3)*
Set threshold to achieve 50% specificity^10,25,27,32,34,35,42,44,48,49,51,58,65^ ^e^	14	4216	19.0%	82.3 (77.8–86.1)	*53.6* (*25.6–81.7)*	49.7 (48.1–51.3)	*0.0* (*0.0–79.9)*

^a^The sensitivity estimate derived based on 70% specificity is used in this analysis when multiple estimates are given and for all other studies, the sensitivity and specificity estimates as reported by authors are used

^b^One study^36^ provides estimates for two genes (EPB41L3 and SOX1); in this analysis only data for EPB41L3 are included so that data from the same population of women are considered only once; similarly for ref. ^38^—data for PAX1 and not SOX1 are included

^c^For analysis of case-control studies, ref. ^10^ was removed from the analysis; and for convenience studies ref. ^49^ was removed from the analysis to allow best model fit in stata

^d^Among women with HPV16 infection; ^e^24 studies allowed standardization of threshold-level for methylation positivity, which corresponded to a specificity of 70% for CIN2+ either by providing the raw data or a ROC curve; 17 studies allowed estimation at a specificity of 50% for CIN2+; 19 studies allowed estimation at a specificity of 70% for CIN3+

^e^14 studies allowed estimation at a specificity of 50% for CIN3+

^f^One study^32^ provides estimates for two gene combinations (CADM1/MAL and MAL/MiR-124-2) and only data for MAL/MiR-124-2 combination were used in the analysis (as authors conclude this was the best combination of markers)

Eleven studies evaluated cell adhesion molecule 1 (*CADM1*), myelin and lymphocyte (*MAL*) and microRNA 124-2 (*MIR*) in different combinations^26,27,32,34,35,45–50^ (Table 1); nine studies evaluated erythrocyte membrane protein band 4.1 like (*EPB41L3*) alone^25,28,29,36,51–53,57^ and three in combination with DNA methylation of HPV16 (L1 and L2 regions), HPV18 (L2), HPV31 (L1) and HPV33 (L2), defined as the S5 classifier (a triage classifier based on DNA methylation of the late regions of HPV16, HPV18, HPV31 and HPV33 combined with the promoter region of a human gene EPB41L3);^10,33,42^ seven for paired box 1 (*PAX-1*) alone,^38–41,43,44,66^ five for sex-determining region Y, box 1 (*SOX-1*) alone^29,36,38,41,44^ and four for family with sequence similarity 19-member A4 (*FAM19A4*), one of which was combined with *MIR-124-2*.^30,31,37,65^ There was one large referral-population-based study evaluating POU Class 4 Homeobox 3 (*POU4F3*) as a single human gene DNA methylation for CIN2+ detection.^24^ Ten studies reported the association of HPV16 (L1 and/or L2) DNA methylation with CIN2+/CIN3+.^33,55–63^ Supplementary Table 3 summarises the  CpG sites targeted for each gene.

**Fig. 2:**
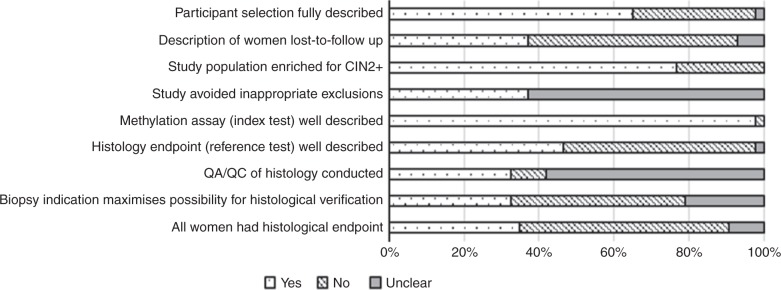
Quality assessment of included studies

The quality of individual studies assessed using QUADAS-2 scores is summarised in Fig. 2, Supplementary Table 1. The majority of included studies were convenience or case-control studies, or among women with HPV16 infection only, and many of these studies had an overrepresentation of women with CIN2+. In 15 (35%) studies, histological verification was available for all women (i.e. colposcopy-directed biopsies were taken when indicated and random biopsies taken from women with normal colposcopy findings), and the remaining studies had histological verification only among women for whom biopsy was indicated following an abnormal colposcopy result (Supplementary Table 2).

### Associations of individual DNA methylation markers with grade of cervical intraepithelial neoplasia grade (*Analysis 1*)

DNA methylation increased with increasing grades of CIN for all DNA methylation markers (Supplementary Fig. 1). Compared to women with ≤CIN1, women with CIN2 had an increased risk of being methylation positive by any of the seven markers (crude OR = 2.83, 95% CI: 2.01–4.00, *I*^2^ = 63%; Supplementary Table 4). Women with CIN3 and ICC were at higher risk of being methylation-positive compared to women with ≤CIN1 (CIN3 vs. ≤CIN1: crude OR = 7.92, 95% CI: 6.10–10.29, *I*^2^ = 43%; ICC vs. ≤CIN1: crude OR = 32.11, 95% CI: 22.51–45.79, *I*^2^ = 0%). When restricting the analysis to women with CIN2 and CIN3 only, the risk of methylation positivity was higher among women with CIN3 compared to women with CIN2 (crude OR = 2.95, 95% CI: 2.03–4.27, *I*^2^ = 71%). This association was observed for all genes, with the exception of *MAL, MIR-124-2* and *POU4F3*, although there was a small number of studies included for these genes.

### Meta-analysis of sensitivity and specificity of DNA methylation markers for the detection of CIN2+ and CIN3+ (*Analysis 2*)

When all gene markers were combined, irrespective of threshold used to define methylation positivity, the pooled sensitivity and specificity estimates for CIN2+ were 63.2% (95% CI: 56.4–69.5) and 75.9% (95% CI: 71.9–79.5), respectively (Table 2, Supplementary Fig. 2**)**. The corresponding estimates for CIN3+ were 70.5% (95% CI: 64.8–75.6) and 74.7% (95% CI: 70.8–78.1; Table 2, Supplementary Fig. 3). A high degree of heterogeneity was observed among studies for both outcomes (Table 2).

When restricting to studies allowing standardisation of specificity at 70%, the pooled sensitivity for CIN2+ and CIN3+ was 68.6% (95% CI: 62.9–73.8) and 71.1% (95% CI: 65.7–76.0), respectively. At a set specificity of 50%, the pooled sensitivity for CIN2+ and CIN3+ was 80.3% (95% CI: 75.6–84.4) and 82.3% (95% CI: 77.8–86.1), respectively.

When results were stratified by study design, sensitivity estimates for CIN2+ were highest for studies focusing on HPV16-positive women (71.8%, 95% CI: 54.5–84.4) with similar high specificity (73.5, 95% CI: 66.6–79.4; Table 2); 8 of these studies evaluated HPV16/L1 methylation^55,56,58–63^ and one study evaluated *EPB41L3*.^57^ For CIN3+, sensitivity was highest for cohort studies (Table 2), although these estimates were not adjusted for gene target, which may have influenced the findings. Assessment of individual gene performance was not possible for each of the target genes but among those that had sufficient number of studies, the sensitivity for CIN2+ detection was highest for HPV16 L1/L2 DNA methylation among women positive for HPV16 (sensitivity: 73.5%, 95% CI: 57.3–85.2; specificity: 72.8%, 95% CI: 65.9–78.7). Among all women, irrespective of HPV16 test positivity, *EPB41L3* and *FAM19A4* had the highest sensitivity (60.2% and 60.0%, respectively) with high specificity (74.6% and 73.0%, respectively; Supplementary Table 5).

### Meta-analysis of positive predictive value of DNA methylation markers for the detection of CIN2+ and CIN3+ (*Analysis 2*)

Based on the observed pooled CIN2+ prevalence of 36.7% among all studies, the pooled PPV for CIN2+ detection was 57.6% (95% CI: 50.3–64.6; Table 3), and this varied according to study design (43.4%, 45.0%. 51.5%, 59.6% and 79.0% for referral-population-based, cohort, case-control, convenience and HPV16-positive women studies, respectively; Model 1; Supplementary Table 6) reflecting the difference in CIN2+ prevalence in these studies. When a fixed (expected) CIN2+ prevalence of 30% was included in the model (Model 2), the pooled PPV was 52.9% (95% CI: 48.4–57.4; Table 3). When restricting analysis to studies reporting threshold to achieve 70% specificity and 50% specificity, the pooled PPV was 38.6% (95% CI: 29.1–48.1), and 40.4% (39.0–41.8), respectively (Table 3). Based on the observed pooled CIN3+ prevalence of 21.6%, the pooled PPV was 40.8% (95% CI: 33.9–48.0), and similarly varied according to study design (Supplementary Table 6**)**.

**Table 3:** Pooled Positive Predictive Value (PPV) of DNA methylation assays for detection of CIN2+ and CIN3+

CIN2+ detection	Model 1^a^	Model 2^b^						
	Observed prevalence	PPV (95% CI)	Set prevalence					
			5%	10%	20%	30%	40%	50%
			PPV (95% CI)	PPV (95% CI)	PPV (95% CI)	PPV (95% CI)	PPV (95% CI)	PPV (95% CI)
All studies (*n* = 38)^10,24–31,33–40,42,43,45–49,52–63,65^	36.7%	57.6 (50.3–64.6)	12.1 (10.2–14.0)	22.6 (19.4–25.7)	39.6 (35.3–43.9)	52.9 (48.4–57.4)	63.6 (59.4.3–67.8)	72.4 (68.8–76.0)
*p-heterogeneity*		*25.1* (*17.1–35.2)*						
Threshold to achieve 70% specificity (*n* = 24 studies)^10,24–29,31,33,35,37,39,42,46,48,55–60,63,65^	35.7%	53.4 (44.4–62.1)	7.2 (4.5–9.8)	14.0 (9.2–18.8)	26.8 (19.0–34.7)	38.6 (29.1–48.1)	49.5 (39.4–59.5)	59.5 (49.8–69.1)
*p-heterogeneity*		*27.5* (*17.1–41.0)*						
Threshold to achieve 50% specificity (*n* = 17 studies) specificity^10,25,27,28,33–35,37,39,42,48,49,55,57,58,60^	34.9%	43.8 (35.2–2.7)	7.7 (7.3–8.1)	15.0 (14.2–15.7)	28.4 (27.2–29.6)	40.4 (39.0–41.8)	51.4 (49.9–52.8)	61.3 (59.9–62.7)
*p-heterogeneity*		*25.5* (*13.8–42.1)*						

CIN3+ detection	Model 1^a^	Model 2^b^						
	Observed prevalence	PPV (95% CI)	Estimated prevalence					
			5%	10%	20%	30%	40%	50%
			PPV (95% CI)	PPV (95% CI)	PPV (95% CI)	PPV (95% CI)	PPV (95% CI)	PPV (95% CI)
All studies (*n* = 30)^10,25–27,29–32,34,35,38,40–42,44,46–54,56,58,62,63,65^	21.6%	40.8 (33.9–48.0)	12.7 (11.0–14.5)	23.6 (20.8–26.3)	41.0 (37.2–44.7)	54.3 (50.5–58.1)	64.9 (61.4–68.4)	73.5 (70.5– 76.5)
*p-heterogeneity*		*17.0* (*10.2–27.9)*						
Threshold to achieve 70% specificity (*n* = 19 studies)^10,25–27,29,31,32,35,41,42,44,46,48,51,53,58,63,65^	19.8%	35.0 (28.9–41.6)	11.0 (10.2–11.8)	20.7 (19.4–22.1)	37.0 (35.2–38.9)	50.2 (48.2–52.2)	61.1 (59.1–63.0)	70.2 (68.5–71.9)
*p-heterogeneity*		*12.6* (*6.0–24.3)*						
Threshold to achieve 50% specificity (*n* = 14)^10,25,27,32,34,35,42,44,48,49,51,58,65^	19.0%	26.5 (21.3–32.4)	8.0 (7.5–8.4)	15.5 (14.7–16.3)	29.2 (27.9–30.4)	41.4 (39.9–42.6)	52.3 (50.8–53.8)	62.2 (60.8–63.6)
*p-heterogeneity*		*14.1* (*5.8–30.4)*						

^a^Model 1: PPV bivariate model from the observed data

^b^Model 2: PPV obtained from pooled specifity and sensitivity by study design at different levels of prevalence of disease, PPV=Prev*SE/(Prev*SE+(1-Prev)*(1-Spec))

### Performance of methylation markers relative to cytology and HPV16/18 genotyping *(Analysis 3)*

In 11 studies, which compared the performance of DNA methylation and cervical cytology among HR-HPV-positive women, DNA methylation assays were marginally less sensitive for CIN2+ and CIN3+ detection compared to cytology ASCUS+ (DNA methylation versus ASCUS+: relative sensitivity = 0.81, 95% CI: 0.63–1.04 [CIN2+]; 0.87, 95% CI: 0.65–1.17 [CIN3+]) but more specific (relative specificity = 1.25, 95% CI: 0.99–1.59 [CIN2+]; 1.37, 95% CI: 1.02–1.85; *p* = 0.04 [CIN3+]). Similarly, relative sensitivity of DNA methylation was lower and relative specificity was higher for CIN2+/CIN3+ when compared to a cytology cut-off of LSIL+, although there were fewer studies (Table 4).

**Table 4:** Pooled relative sensitivity and relative specificity of DNA methylation assays compared to cytology and HPV16/18 genotyping for detection of CIN2+ and CIN3+ following a HR-HPV-positive result, and compared to HR-HPV DNA test

	CIN2+^a^					CIN3+^b^				
	*N* studies	Relative sensitivity (95% CI)	*p*-value	Relative specificity (95% CI)	*p*-value	*N* studies	Relative sensitivity (95% CI)	*p*-value	Relative specificity (95% CI)	*p*-value
*Among HR-HPV-positive women*^c^										
Cytology ASCUS+^10,24–26,31,35,47,51,55,65^	11	0.81 (0.63–1.04)	0.102	1.25 (0.99–1.59)	0.116	9	0.87 (0.65–1.17)	0.351	1.37 (1.02–1.85)	0.037
Cytology LSIL+^25,35,44,46^	4	0.65 (0.40–1.08)	0.095	1.55 (0.92–2.61)	0.096	5	0.71 (0.61–0.82)	<0.001	1.36 (0.69–2.68)	0.371
HPV16/18 genotyping^e^^10,25,27,30,31,35,36,42^	9	1.22 (1.05–1.42)	0.01	1.03 (0.94–1.13)	0.552	8	1.19 (0.97–1.45)	0.091	1.04 (0.97–1.12)	0.234
*Among all women irrespective of HR-HPV status*^d^										
HR-HPV DNA^25,34,36,40,43,44,46,47,52,54^	10	0.58 (0.47–0.72)	<0.001	1.63 (1.30–2.05)	<0.001	9	0.71 (0.61–0.83)	<0.001	1.66 (1.23–2.25)	0.001

Twelve studies^10,24–26,31,35,44,46,47,49,51,55,65^ evaluated cytology (abnormalities defined as ASCUS+ in ten studies, LSIL+ in five studies) following a HR-HPV test or among women with high prevalence of HR-HPV; nine^10,25,27,30,31,35,36,42^ evaluated HPV16/18 genotyping following a HR-HPV-positive test; ten studies^25,34,36,40,43,44,46,47,52,54^ evaluated performance of HR-HPV testing (any type positive using Hybrid Capture II or genotyping methods); to ensure standardisation of the raw data across all of the three analyses, we used sensitivity estimates based on a set specificity of 70%, where available

^a^vs. ≤CIN1

^b^vs. ≤CIN2

^c^Test evaluated among women with HR-HPV-positive test result

^d^Test evaluated among all women, irrespective of HR-HPV DNA status

^e^In the analyis comparing DNA methylation versus HPV16/18 genotyping, the combination of DNA methylation assays included human genes only

In nine studies which directly compared DNA methylation and HPV16/18 genotyping among HR-HPV-positive women, DNA methylation assays were significantly more sensitive than HPV16/18 genotyping for CIN2+ (relative sensitivity = 1.22, 95% CI: 1.05–1.42 *p* = 0.01) with similar specificity (relative specificity = 1.03, 95% CI: 0.94–1.13).

In 10 studies that evaluated DNA methylation assays compared to HR-HPV DNA cocktail screening tests, methylation assays were significantly less sensitive for CIN2+ (relative sensitivity = 0.58, 95% CI: 0.47–0.72; *p* < 0.001) but had significantly higher specificity (relative specificity = 1.63, 95% CI: 1.30–2.05; *p* < 0.001).

## Discussion

This meta-analysis investigating the performance of DNA methylation of human genes and HPV virus for the detection of CIN2+ and CIN3+ indicates that DNA methylation of several human genes and HPV16 L1/L2 increased with increasing CIN grade, with significantly higher methylation in CIN3 compared to CIN2, and almost universally high methylation in ICC, confirming the relevance of these markers as potentially useful in the screening and triage settings for the most advanced lesions.

At an expected CIN2+ and CIN3+ prevalence of ~30 and 20%, equivalent for example to a referral-population of women with a HR-HPV-positive test,^27,33^ DNA methylation assays had marginally lower sensitivity for CIN2+ detection and higher specificity compared to cytology (ASCUS+ or LSIL+) and higher sensitivity compared to HPV16/18 genotyping for a similar specificity. Although there were too few studies to conduct a discrete meta-analysis, the S5 classifier had higher sensitivity for CIN2+ detection compared to the human gene *EPB41L3* alone without compromising specificity,^33,42^ suggesting the combination of viral and host cell targets may improve accuracy to detect CIN2+. Future studies may evaluate methylation of a wider range of HPV types found to be associated with CIN2+.

An optimal triage test should have high sensitivity to ensure women with confirmed high-grade lesions receive appropriate management and high PPV to ensure women who test positive are accurately targeted for management, avoiding overtreatment, associated costs and patient anxiety. In our review, DNA methylation assays generated a pooled PPV of 53 and 35% for CIN2+ and CIN3+, with corresponding sensitivity of 69 and 71%, respectively. These estimates are similar to that reported for cytology ASCUS+ among 535 HR-HPV-positive women enrolled in a population-based screening study in the Netherlands,^67^ that reported PPV and sensitivity of 60 and 63%, respectively, for CIN2+ and 42 and 71%, respectively, for CIN3+. The corresponding estimates for HPV16/18 genotyping were 38 and 59%, respectively, for CIN2+, and 26 and 65%, respectively, for CIN3+. Among 614 HR-HPV-positive women participating in the Canadian Cervical Cancer Screening Trial,^68^ cytology ASCUS+ had a lower PPV of 30% with a low sensitivity (48%) for CIN2+, while HPV16/18 genotyping had a PPV and sensitivity of 32 and 64%, respectively. There were too few prospective studies evaluating DNA methylation markers to conclusively assess their potential as predictors of future or progressing cervical lesions. However, three recent studies highlight their potential usefulness in that regard. A longitudinal study among 1040 HPV-positive women enrolled in the POBASCAM screening trial in the Netherlands reported that, compared to a cytology negative (<ASCUS) result at enrolment, a negative *FAM19A4/MIR124-2* methylation test indicated lower risk of cervical cancer incidence over a 14-year follow-up period (Risk Ratio = 0.74, 95% CI: 0.16–1.40).^69^ In a cohort of women living with HIV in South Africa, participants with persistent CIN3, or CIN2 which progressed to CIN3, had significantly higher baseline *EPB41L3* methylation levels compared to women who remained ≤CIN1 over 16 months, and compared to women with spontaneous regression to ≤CIN1.^25^ In a study among 149 women with CIN2 that were followed over 2 years in Finland, the S5-classifier had the highest sensitivity to predict CIN2 lesions that progressed to CIN3 from those that spontaneously regressed to ≤CIN1 compared to cytology (using various cut points), HPV16/18 or HPV16/18/31/33 genotyping.^70^

In comparison to other triage tests such as cytology and p16^INK4A^ staining, the advantages afforded by DNA methylation assays are that their molecular basis makes them automatable and less prone to training and interpretational errors than the morphological tests. Testing can be performed using the same clinician-collected or self-collected sample used for HPV screening,^30^ thereby simplifying sample collection. Methylation could therefore become a useful alternative to cytology as a triage test among HR-HPV-positive women. Moreover, methylation assays provide an advantage over HPV16/18 genotyping as they are not restricted to detection of CIN2+ associated with HPV16/18 only, combining a higher sensitivity for CIN2+ with a similar specificity. While current methylation technologies may not yet be suitable for low-resource settings, technological advances may allow for use in such settings in the not too distant future.

There were too few studies in our review allowing for an evaluation of DNA methylation assays as a primary screening test. However, eleven studies evaluating human genes DNA methylation assays among populations with high HR-HPV prevalence have shown that these assays had higher specificity compared to primary HPV DNA screening, albeit with lower sensitivity. Assays combining human genes and HPV viral methylation may therefore increase sensitivity for CIN2+ detection while retaining high specificity, a useful feature in populations with high prevalence of HR-HPV. Given the potential for self-sampling, this approach may allow for a one-sample one-visit screening, which would reduce the loss-to-follow-up of women in many low-resource settings where HR-HPV prevalence may be high and where access to screening may be limited, allowing the number of screening visits in a woman’s lifetime to be reduced. It is important, however, that any recommendations for inclusion of methylation tests in screening or triage will have to consider affordability, cost-effectiveness and ease of management.

There were a number of limitations to our review. Firstly, there was significant heterogeneity in the pooled performance estimates, which may be explained by any of the following: (1) variability in study designs; (2) variability in the proportion of CIN2+ cases included in each study; (3) differences in the target genes and CpG sites studied and (4) variation in thresholds used to define methylation positivity. We sought to limit the effects of these variations in our analysis. We stratified performance estimates by study design to distinguish the performance of DNA methylation in studies that are in an early discovery phase (i.e. mostly convenience and case-control studies) from those studies focused on defining the performance of these markers for screening or triage in referral-population-based and cohort studies. In order to adjust for differences in methylation threshold levels, we derived pooled sensitivity from those studies that allowed us to set specificity at 70%. Where possible, we obtained pooled sensitivity for individual target genes that revealed differences in sensitivity, with higher sensitivity achieved with combination markers compared to individual genes. Because PPV estimates correlate with prevalence of disease, we observed heterogeneity in the PPV estimates, largely due to the variability in the proportion of CIN2 cases included in each study. We controlled for this variability by generating a pooled PPV for different fixed levels of CIN2+ and CIN3+. We assumed no change in performance of DNA methylation assays with increasing prevalence of CIN2+, although future studies may demonstrate changes in sensitivity and/or specificity for CIN2+, depending on gene target as we currently see for HR-HPV DNA-based tests. Second, this review may have some selection bias, as we limited ourselves to include the most widely studied target genes, and a minimum number of reports for each gene. There was clear overrepresentation of women enrolled in large studies in the Netherlands (PROHTECT and POBASCAM) and the UK (PREDICTORS-1 and -2), as these groups have been most active in this particular field. The associations of individual gene marker methylation with increasing CIN grades is limited by the low number of studies for several gene targets included in the analysis, and we were unable to present adjusted estimates. Finally, not all studies (35% of studies only) had histological endpoints for all women included in the analysis, as biopsy indication was often based on colposcopy findings, leading to some disease misclassification linked to the variable sensitivity of cytology and colposcopy.^71^

In conclusion, DNA methylation assays show promise for the detection of CIN2+ in triage situations, combined with existing screening tools with high sensitivity but lower specificity, such as HPV DNA tests. Methylation could be a useful alternative to cytology as a triage test among HR-HPV-positive women, given their similar performance with the added advantages of objectivity, automation and self-collected sampling. Despite an increasing number of studies in recent years evaluating different gene targets, the strength of current evidence remains low, and randomised controlled trials and further large prospective studies following guidelines on rigorous biomarker evaluation^72^ are needed.

## Supplementary information

Supplementary information

## Data Availability

Data are available at Mendeley (10.17632/84khm3rf8k.1).
