# Supplementary material for: Performance of DNA methylation assays for detection of high-grade cervical intraepithelial neoplasia (CIN2+): a systematic review and meta-analysis
Source: Br J Cancer. 2019 Oct 16;121(11):954–65. doi: 10.1038/s41416-019-0593-4 (PMC6889421; doi:10.1038/s41416-019-0593-4)
Supplement: Supplementary file 1 — Supplementary information [file 41416_2019_593_MOESM1_ESM.docx]

**Supplementary Table 1. Quality assessment of included studies**

| **Author, year** | **StudyID** | **Risk of bias** | | | | | | | | | | | | **Concerns of applicability** | | |
| --- | --- | --- | --- | --- | --- | --- | --- | --- | --- | --- | --- | --- | --- | --- | --- | --- |
|  |  | **Patient selection** | | | **Index test** | | | **Reference standard** | | | **Flow and timing** | | | **Patient selection** | **Index test** | **Reference test** |
|  |  | **P1** | **P2** | **P3** | **T1** | **T2** | **T3** | **R1** | **R2** | **R3** | **F1** | **F2** | **F3** |  |  |  |
| **Referral population based studies** |  |  |  |  |  |  |  |  |  |  |  |  |  |  |  |  |
| Verhoef, 2015 | C6 | Y | N | Y | Y | Y | Y | Y | Y | Y | Y | Y | Y | Low | Low | Low |
| Verhoef, 2014 | C9 | Y | N | Y | Y | Y | Y | Y | Y | Y | Y | U | Y | Low | Low | Low |
| Hesselink, 2014 | C10 | Y | Y | U | Y | Y | Y | Y | U | Y | N | Y | N | Low | Low | Low |
| DeStrooper, 2014 | F1 | Y | Y | U | Y | Y | Y | Y | Y | Y | U | U | Y | Low | Low | Low |
| Luttmer, 2016 | F2 | Y | Y | U | Y | Y | Y | Y | U | Y | Y | Y | Y | Low | Low | Low |
| Kocsis, 2016 | P1 | Y | N | Y | Y | Y | Y | N | U | N | Y | U | N | Low | Low | Possible |
| Vasiljevic, 2014 | E4 | Y | Y | U | Y | N -ROC | Y | Y | Y | N | Y | U | N | Low | Low | Low |
| Van Leeuwan, 2018 | E7 | Y | N | Y | Y | Y | Y | N | Y | N | N | Y | N | Low | Low | Possible |
| Brentnall, 2014 | H16-6 | Y | Y | Y | Y | N -ROC | U | Y | Y | N | N | U | N | High | Low | Low |
| **Cohort studies** |  |  |  |  |  |  |  |  |  |  |  |  |  |  |  |  |
| Van Zummern, 2017 | C2 | Y | N | Y | Y | Y | Y | Y | U | Y | Y | Y | Y | Low | Low | Low |
| De Vuyst, 2015 | C5 | Y | N | Y | Y | Y | Y | Y | U | Y | Y | Y | Y | Possible | Low | Low |
| Rogeri, 2018 | E11 | N | Y | U | Y | Y | U | N | U | N | N | U | N | High | Low | High |
| Bu, 2018 | F4 | Y | Y | U | Y | N -ROC | Y | N | U | Y | Y | Y | N | High | Low | Possible |
| Kan, 2014 | PS2 | Y | N | Y | Y | Y | Y | N | U | N | N | Y | N | Low | Low | High |
| Li, 2015 | PS4 | Y | N | Y | Y | N -ROC | Y | N | Y | U | U | U | N | Low | Low | Possible |
| Lin, 2011 | PS5 | Y | Y | U | Y | U | Y | Y | Y | N | Y | U | N | High | Low | Possible |
| Tian, 2017 | PS8 | Y | N | Y | N | Y | Y | N | N | N | N | Y | N | Low | Low | High |
| **Case-control studies** |  |  |  |  |  |  |  |  |  |  |  |  |  |  |  |  |
| Kelly, 2018-BF | E1 | Y | Y | Y | Y | Y | Y | Y | Y | Y | Y | Y | Y | Possible | Low | Low |
| Kelly, 2018-SA | E1 | Y | Y | Y | Y | Y | Y | Y | Y | Y | Y | Y | Y | Possible | Low | Low |
| Lorincz, 2016 | E2 | Y | Y | Y | Y | Y | Y | Y | N | Y | Y | Y | Y | Possible | Low | Low |
| Cook, 2018 | E8 | Y | N | Y | Y | N - ROC | Y | Y | Y | Y | N | Y | Y | Possible | Low | Low |
| Xu, 2015 | PS1 | N | Y | U | Y | N -ROC | Y | N | U | U | N | U | U | High | Low | Possible |
| Lai, 2014 | PS3 | N | Y | U | Y | N -ROC | Y | N | U | N | N | U | U | High | Low | Possible |
| **Convenience studies** |  |  |  |  |  |  |  |  |  |  |  |  |  |  |  |  |
| Kim, 2016 | C3 | N | Y | U | Y | Y | Y | N | U | N | N | U | N | High | Low | High |
| Van Baars, 2016 | C4 | N | Y | U | Y | Y | Y | Y | Y | Y | Y | Y | Y | High | Low | Low |
| De Strooper, 2014 | C7 | N | Y | U | Y | Y | Y | N | U | U | N | U | N | High | Low | Possible |
| De Strooper, 2014 | C8 | Y | Y | U | Y | Y | Y | N | U | N | N | U | N | High | Low | Possible |
| Hesselink, 2011 | C11 | Y | Y | U | Y | Y | Y | N | U | N | N | U | N | High | Low | Possible |
| Overmeer, 2011 | C12 | U | Y | U | Y | Y | Y | N | U | N | N | U | N | High | Low | Possible |
| Boers, 2014 | E5 | Y | Y | U | Y | N -ROC | Y | Y | Y | Y | N | U | Y | High | Low | Low |
| Eijinsk, 2012 | E6 | N | Y | U | Y | Y | Y | U | U | U | N | U | U | High | Low | Possible |
| Boers, 2016 | E9 | N | Y | U | Y | Y | U | Y | N | N | N | U | N | High | Low | Possible |
| DeStrooper, 2014 | F3 | N | Y | U | Y | Y | Y | N | U | N | N | U | N | High | Low | Possible |
| Huang, 2010 | PS6 | N | Y | U | Y | Y | Y | Y | N | N | N | U | N | High | Low | High |
| **Studies among women with HPV16 infection** |  |  |  |  |  |  |  |  |  |  |  |  |  |  |  |  |
| Louvanto, 2016 | E3 | Y | Y | U | Y | Y | Y | Y | U | N | U | U | N | High | Low | Possible |
| Bryant, 2016 | H16-1 | Y | Y | U | Y | N -ROC | Y | N | U | N | Y | U | N | High | Low | High |
| Mirabello, 2015 | H16-2 | N | Y | U | Y | N -ROC | U | N | U | N | N | U | U | High | Low | Possible |
| Qiu, 2015 | H16-3 | N | Y | U | Y | Y | U | N | Y | N | N | U | N | High | Low | High |
| Simanaviciene, 2015 | H16-4 | N | Y | U | Y | U | U | N | U | U | N | U | Y | High | Low | Possible |
| Brandsma, 2014 | H16-5 | N | Y | U | Y | Y | Y | N | U | U | N | U | Y | High | Low | Possible |
| Lorincz, 2013 | H16-7 | Y | Y | Y | Y | Y | U | Y | U | U | Y | U | Y | High | Low | Possible |
| Mirabello, 2013 | H16-8 | Y | Y | U | Y | N -ROC | U | N | U | U | N | U | Y | High | Low | Possible |
| Kottaridi, 2017 | H16-9 | Y | Y | Y | Y | Y | U | N | U | U | Y | U | Y | High | Low | Possible |

QUADAS items: P1= participant selection fully described; P2= study population enriched for CIN2+ (in context of triage setting [HR-HPV positive or ASCUS+] among women of screening age, expected CIN2+ prevalence following HR-HPV positive test=20-30%; in cohort/screening setting, an expected CIN2+ prevalence <10% among HIV negative women and 10-25% among women living with HIV); P3= study avoided inappropriate exclusions; T1= Index test well described; T2= threshold for methylation positivity prespecified (ROC indicates if determined from Receiver Operating Curve) ; T3= Index test was conducted and interpreted without knowledge of reference test result; R1= Reference test well described; R2= QA/QC of reference test conducted; R3= Biopsy indication maximises possibility for histological verification (misclassification bias); F1=Description of women lost-to-follow up; F2= Appropriate interval between index and reference test; F3= All women had same reference test (i.e. all women had histological verification of CIN); Y=fulfilled; N=not fulfilled; U=Unclear

**Supplementary Table 2. Description of included studies for systematic review of DNA methylation and CIN2+ /CIN3+**

| **Author, year** | **Study ID** | **Population** | **Biopsy indication and histological verification** | **Methylation assay** |
| --- | --- | --- | --- | --- |
| ***Referral population based studies*** | | | | |
| Verhoef, 2015 | C6 | **HPV positive** women from Dutch screening programme - PROHTECT-3 RCT 2010-2011 (analysis of Cytology arm), cytology triage group, self-samples [non-responder population]. Exclusion of previous hysterectomy, or CIN2+ or abnormal cytology in previous 2 yrs. All women from this group with HPV+ result and valid sample for testing. | Women with HR-HPV positive test and positive triage test (cytology or methylation as part of RCT) were indicated for colposcopy directed biopsy. Cervical abnormalities were biopsied and treated according to the standard protocol in the Netherlands. Two random biopsies at twelve o'clock and six o'clock were advised in women with a normal colposcopic impression.  Women with normal cytology were offered an exit test after 6 months, which consisted of cytology and HR-HPV co-testing. At that occasion, women were referred for colposcopy if any of both tests was abnormal (i.e., abnormal cytology and/or HR-HPV positive). Women with double negative results (i.e., normal cytology and HR-HPV-negative) were sent back to regular screening.  All women with a double negative exit test or a histological outcome before the end of the study (i.e. January 2013) reached the study endpoint. Women without a study-endpoint at January 2013, which is at least 12 months after entering the study, were labelled ‘no CIN2+detected’.  Histology was read according to current guidelines in different pathology laboratories and results were registered in the nationwide histopathology and cytopathology registry in the Netherlands.  **Low risk** | QMSP |
| Verhoef, 2014 | C9 | PROHTECT-3 RCT 2010-2011 (analysis of cytology AND methylation arms).  **As for C6**  **Low risk** | As for C6  **Low risk** | QMSP |
| Hesselink, 2014 | C10 | Non-attending women from Dutch screening programme who were HR-HPV positive (PROHTECT-1 STUDY 2005) using self-samples. | Women who were HR-HPV positive and cytology abnormal (i.e., ASCUS+) were referred for colposcopy-directed  biopsy. HR-HPV positive women with normal cytology received an advice for repeat co-testing (cytology and HR-HPV) after 1 year and were referred for colposcopy-directed biopsy in case of abnormal cytology, a positive HR-HPV test, or both.  **Low risk (of misclassification bias if HR-HPV negative and cytology normal**) | QMSP |
| DeStrooper, 2014 | F1 | PROHTECT-3 RCT 2010-2011 (analysis of methylation arm)  As for C6  **Low risk** | As for C6  **Low risk** |  |
| Luttmer, 2016 | F2 | Women who were HR-HPV positive recruited in prospective multi-centre cohort study, recruited from gynaecological outpatient clinics (COMETH study). | HR-HPV positive women referred for colposcopy. Cervical biopsies were taken from every visible lesion for histological assessment. In case no lesions were visible, it was mandatory to take two random biopsies (6 and 12 o’  clock). Endocervical curettage taken if squamocolumnar junction not visible.  **Low risk** | QMSP |
| Kocsis, 2016 | P1 | Women recruited from 4 sites in Hungary as part of the Triage and Risk Assessment of Cervical Precancer by Epigenetic Biomarker (TRACE) prospective, multicentre study which aimed to provide a clinical evaluation of the CONFIDENCE^TM^ assay. All women underwent computer-assisted VIA followed by LBC sampling for cytology and HPV; methylation assay evaluated among HR-HPV+ as triage test. | Women attending outpatient clinics were screened and followed-up according to the Hungarian guidelines; liquid based cytology sampling was performed followed by colposcopy-assisted visual inspection of the cervix with acetic acid test (VIA). Depending on the result of VIA, LBC and HPV detection gynaecologists made medical decisions and if necessary referred these women for cone or punch biopsy.  Women attending oncology centres had LBC taken prior to the intervention (cone biopsy or hysterectomy). Not all  patients of the oncology centre were referred by one of the four outpatient clinics and likewise, several patients were not referred and were treated by the outpatients clinics  **High risk**, unclear biopsy indication and pathology classification (possible misclassification bias) | qMSP (CONFIDENCE Biomarker^TM^) |
| Vasiljevic, 2014 | E4 | HR-HPV positive women, Colposcopy referral sample, referred due to abnormal smear (PREDICTORS-1&-2 studies)  Study population comprised 1,099 women who had been referred to the colposcopy clinics at the Hammersmith and St. Mary's Hospitals in London, United Kingdom, between September 2007 and October 2009 because of abnormal screening smears. | Women attending colposcopy due to abnormal cytology (ASCUS+). Histopathology was first reported locally and then centrally reviewed by an independent pathologist, who was blinded to all study test results. Where discrepant readings occurred, further review was undertaken, and the majority opinion was taken; where all three pathologist readings were discrepant (one each of CIN less severe than grade 2 [<CIN2], CIN2, and CIN3), CIN2 was assigned.  All results are presented on the basis of the reviewed histopathology, and the highest grade of abnormality seen in the biopsy or treatment specimen was used.  Unclear if biopsy indicated based on colposcopic abnormality.  **Low-Medium risk** | PSQ |
| Van Leeuwan, 2018 | E7 | Prospective population-based cohort study - women participating in the Slovenian HPV Prevalence Study were eligible for inclusion if they attended routine organised Slovenian national cervical cancer screening program. Women were eligible if they tested positive in the Abbott RealTime High Risk HPV assay. | Not detailed but referred to previous paper^[1]^:  According to the criteria of the Slovenian National Cervical Cancer Screening Program, women were called for immediate colposcopy if cytology ASCUS+. In addition, irrespective of their cytology result, according to study protocol, women were also invited for colposcopy if they were positive for HPV16 or HPV18. In women  positive for HR-HPV other than HPV16 and HPV18, immediate colposcopy was performed at the physician’s discretion; otherwise, the woman was invited to a control gynaecological examination after 6 months to 1 year.  During colposcopy, punch biopsy specimens were taken from any regions suspicious for CIN. No biopsy specimen was taken from women with normal colposcopy results. The most severe abnormality was selected for final  histopathological diagnosis.  An expert histopathology system was used for histopathological assessment: all biopsy specimens were first examined by a certified pathologist with more than 20 years of experience in gynaecological pathology, followed by an independent, blinded histopathological review. In discrepant cases, the final diagnosis was the consensus reached by a panel of three pathologists. Pathologists performing histopathological assessments were blinded to the  HPV status but did have access to concurrent cytology results.  **Low-medium risk (misclassification bias of colposcopy normal?)** | QMSP |
| Brentnall, 2014 | H16-6 | PREDICTORS11&2 studies  As for E4, restricted to women with HPV16 infection. | As for E4, restricted to women with HPV16 infection | PSQ |
| **Cohort studies** | | | | |
| Van Zummern, 2017 | C2 | Women living with HIV attending a gynaecological outpatient clinic at Steve Biko Academic Hospital or Tshwane District Hospital Pretoria, South Africa. Almost all women on ART (99%) with median CD4+ cell count of 514 [IQR: 380-720]; HR-HPV positivity=42% | All women referred to colposcopy; two mandatory cervical biopsies were taken from either the most abnormal area or at random (6 and 12 o`clock) if no lesion visible. Endocervical curettage taken if squamo-columnar junction not visible.  No information on histopathological review.  **Low-medium risk** | qMSP (PreCursor-M) |
| De Vuyst, 2015 | C5 | Women living with HIV who were HR-HPV positive recruited as part of a cervical screening study at Coptic Hope Center for Infectious Diseases, Nairobi, Kenya. | All women underwent colposcopic examination and biopsy taken from all women, either from the most abnormal area on the cervix or at 12 o’clock if no lesion was visualized. Conventional cytology slides and biopsies were  read by the study pathologist at the Aga Khan University, Nairobi.  No information on histopathological review  **Low-medium risk** | QMSP |
| Rogeri, 2018 | E11 | Study includes 447 women who underwent colposcopy in the Prevention Department of the Barretos Cancer Hospital from 2014 to 2015. The cases were identified using the database belonging to the Department. | Cervical biopsies or conization (when performed) were considered the gold standard techniques. When biopsy and conization yielded divergent results, the worst diagnosis was assigned. Women with a satisfactory colposcopy without findings that justified cervical biopsy were classified as negative for cervical cancer.  Unclear referral to colposcopy, biopsy indication and histology verification.  **High risk** | QMSP |
| Bu, 2018 | F4 | Patients enrolled in HPV genotyping testing at Guangdong Women and Children Hospital between 2016 and 2017 were purposely selected. Patients with ASCUS+ and HR-HPV positive or HPV16/18 positive or clinical examination of suspected abnormality or suspected history and signs were referred for colposcopy. Biopsies and/or endocervical curettage were taken from abnormal cervical areas during colposcopic examinations. | Women referred to colposcopy if abnormal cytology (ASCUS+) AND HR-HPV positive OR HPV16/18 positive OR clinical exam suggests suspected abnormalities. Biopsy and/or endocervical curettage taken from abnormal areas.  No information on histopathological review  **Low-medium risk** | MSP-qPCR |
| Kan, 2014 | PS2 | Women with normal uterine cervix and patients with abnormal pap results of different stages were recruited from the Yuan's General Hospital. Women with history of reproductive tract related cancer, those who received treatment for cervical lesions or HPV vaccination and pregnant women were excluded. | Women with low- and high-grade lesions, as determined by cytology, underwent colposcopic cervical biopsy.  In the abnormal Pap test result group, the final diagnosis was made by tissue-proven pathology,  Unclear referral to colposcopy, biopsy indication and histology verification.  **High risk** | QMSP |
| Li, 2015 | PS4 | Women participating in screening programme at Weifang city people’s hospital and with an ASC-H diagnosis. Women with history of cancer, women who were immunocompromised or pregnant women were excluded. | All women with cytology ASCUS+ were referred for colposcopy and cervical biopsy. Cervical biopsies were cut and stained with haematoxylin and eosin, read by a pathologist, and confirmed by a second independent reading result.  Unclear if biopsy indicated by colposcopy findings or if all women had biopsy taken.  **Low-medium risk** | MS-HRM |
| Lin, 2011 | PS5 | Women with ASCUS nested in multicentre study attending 11 medical centres in Taiwan. | Women underwent colposcopy with biopsy indicated if colposcopy abnormal (17% of all women indicated for biopsy).  For a quality control of diagnosis, slides of cytology and histology were reviewed by 2 expert cytologists and pathologists, respectively. The diagnoses in cytology and pathology were validated with the National Cervical Cytology and Histology Registry and National Cancer Registry of Taiwan.  **Medium risk (misclassification bias of colposcopy normal women)** | MSP |
| Tian, 2017 | PS8 | Women attending cervical screening who were HR-HPV-positive and negative for HPV 16/18. | All women underwent colposcopy with biopsy indicated if colposcopy abnormal.  Possible misclassification bias of colposcopy normal, no review of histology.  **Medium-High risk**. |  |
| **Case-control studies** | | | | |
| Kelly, 2018  (Burkina Faso & South Africa) | E1 | Women living with HIV recruited in an evaluation study of cervical cancer screening strategies. Case-control study of WLHIV aged 25-50 with histology-determined CIN2+ (cases, N=152) and ≤CIN1 (controls, N=210) ; HR-HPV was 82.8% in Burkina Faso and 86.9% in South Africa. | Systematic 4-quadrant cervical biopsy, including directed biopsy of any suspicious lesions, was performed for participants who had abnormalities detected by cytology (Pap smear), VIA/VILI or colposcopy, or who were HR-HPV DNA positive (HC-II or careHPV). Histology was classified as ‘negative’ (≤CIN1) or ‘positive’ (CIN2+) based on the highest reading across all findings from the 4-quadrant biopsies and endocervical curettage if collected. All histological slides from women with a local diagnosis of CIN2+ and approximately 10% of slides from women with ≤CIN1 histological findings were reviewed by the HARP Endpoint Committee of five pathologists, for consensus classification, which showed high agreement  **Low risk** | PSQ |
| Lorincz, 2016 | E2 | HR-HPV positive samples randomly selected from UK screening group (selection based on HR-HPV positivity, cytology results and CIN status) from PREDICTORS-3 study. | Residual material was used from the liquid-based cytology PreservCyt samples from 6000 women who attended for a routine  3 or 5 yearly (depending on age) screening smear, and whose samples were sent to the cytology laboratory at St. Mary’s Hospital,  London^[2]^. Samples were linked to concurrent cytology results and any histology within 6 months of an abnormal smear.  All results are presented based on the local histopathology and the highest grade of abnormality seen in the biopsy or treatment specimen was used.  It was not possible to undertake histology review, although previous studies (Szarewski et al, 2012) show that when pathology review is possible, 5% of biopsies read as CIN2+ are downgraded to <CIN2; conversely, 6% of biopsies reported as <CIN2 are upgraded to CIN2+ following pathology review.  **Low-medium risk** | PSQ |
| Cook, 2018 | E8 | Blinded case-control study within the HPV FOCAL randomized cervical cancer screening trial of women aged 25-65. Groups were randomly selected from 257 women with known HPV (determined by HC-II)/cytology results and pathology outcomes. Group 1: 104 HPV+, abnormal cytology (54 CIN2/3; 50 <CIN2); Group 2: 103 HR-HPV+, normal cytology with HPV persistence at 12 months (53 CIN2/3; 50 <CIN2); Group 3: 50 HR-HPV+, normal cytology with HR-HPV clearance at 12 months (assumed <CIN2). CIN diagnosis based on histopathology. | HR-HPV (HC-II) positive women were triaged by LBC and referred for colposcopy if abnormal cytological findings. Colposcopy included biopsy and/or endocervical triage for all women. Pathology laboratories where histology was reviewed are affiliated with the British Columbia Cancer Agency  **Low risk** | PSQ |
| Xu, 2015 | PS1 | Women referred for colposcopy or known invasive cervical cancer at the central Hospital of Minhang District. Healthy women were included as healthy controls with cytology (Pap test) done at same time; HPV positive=76% | Women referred to colposcopy (cases) matched with healthy women attending routine screening (controls) had cervical scraping and cervical tissues collected (unclear if all women had biopsy).  **High risk** | PSQ |
| Lai, 2014 | PS3 | Women aged ≥20y attending 11 medical centres in Taiwan, referred for low and high grade lesions identified by cytology and underwent biopsy; controls recruited from healthy women who underwent routine pap screening; final diagnosis made by histopathology for cases and cytology for controls; HR-HPV=44.5% | Patients with low or high grade lesions, as determined by cytology underwent colposcopic cervical biopsy.  No information on histopathological review  **Medium-high risk** | QMSP |
| **Convenience studies** | | | | |
| Kim, 2016 | C3 | Samples collected at Cheil General Hospital & Women's Healthcare Centre; HPV positive with concordant biopsy and cytology (Pap test) result; biopsy-matched LBC sample and not population based screening. | All women had cervical biopsy performed, except 17% of women who had cytology normal or ASCUS.  No information on histopathological review  **High risk**. | PSQ |
| Hesselink, 2011 | C11 | Cervical scraping of first 250 HR-HPV positive women derived from a group of 3,135 women participating in a population based screening programme. | Samples obtained from population based randomised controlled screening POBASCAM trial.  Women with ASCUS+, or (ASCUS and HR-HPV) referred to colposcopy. Colposcopy directed biopsies taken for histology according to standard protocol in the Netherlands.  **Medium risk (misclassification bias)** | QMSP |
| Overmeer, 2011 | C12 | Random selection of 40 cervical scrapings HR-HPV positive, normal cytology with no evidence of CIN disease and 30 scrapings HR-HPV positive with moderate dyskaryosis or worse of HR-HPV positive women with CIN3+ obtained from population-based cervical screening trial POBASCAM. | Samples obtained from population based randomised controlled screening POBASCAM trial.  As for C11  **Medium risk (misclassification bias)** | QMSP |
| Van Baars, 2016 | C4 | Women aged ≥17y referred for colposcopy to Hospital Clinic, Barcelona because of abnormal cervical cytology. Random selection of 70 women with varying grades of CIN, irrespective of HPV status. | Colposcopy performed for all women. During colposcopy, up to four biopsies were collected from different abnormal areas or different regions in one large complex abnormal area of the cervix. If less than four colposcopically directed biopsies were taken, a biopsy from normal appearing epithelium (visually negative tissue) was added. An endocervical curettage (ECC) was performed if clinically indicated.  All biopsies were reviewed by a second independent gynaecological pathologist. In case of disagreement between the original and review diagnosis, a third pathologist reviewed the discordant cases independently and a consensus diagnosis was reached by agreement between two of three interpretations. All pathologists were blinded for HPV status and cytology result. The overall histological diagnosis per women was based on the worst diagnosis found in all biopsy specimens of each woman.  **Low risk** | qMSP (PreCursor-M prototype) |
| De Strooper, 2014 | C7 | Women participating in population based screening or attending gynaecological outpatient clinic [79 ICC, 16 CIN3, 32 CIN2; 120 <CIN2] ; a higher than normal proportion of HPV-positive (80%) were selected. | Unclear referral to colposcopy, biopsy indication and histology verification.  **High risk** | qMSP (PreCursor-M) |
| De Strooper, 2014 | C8 | Women participating in population-based screening (similar to POBASCAM trial) who were HR-HPV positive. | Samples obtained from population based randomised controlled screening POBASCAM trial.  As for C11  **Medium risk (misclassification bias)** | qMSP |
| Boers, 2014 | E5 | Sample of women selected from PROHTECT-3B (physician collected arm)  As for C6. | As for C6.  **Low risk** | QMSP |
| Eijinsk, 2012 | E6 | Patients referred to colposcopy with abnormal smear irrespective of HR-HPV | All women referred with abnormal smear were diagnosed by biopsy or large loop excision of transformation zone.  **Medium risk (misclassification bias)** | QMSP |
| Boers, 2016 | E9 | Patients referred to the outpatient clinic of the University Medical Center Groningen (UMCG) with cervical cancer or an abnormal Pap smear at population-based screening. The histological classifications of these patients were 27 without CIN, 38 CIN1, 45 CIN2, 61 CIN3, and (29 squamous cell carcinoma, 12 adenocarcinoma, 3 adenosquamous carcinoma). | Women attending population based screening referred to colposcopy with abnormal cytology smear.  At colposcopy, biopsies and/or large loop excision of the transformation zone (LLETZ) were performed. The tissue samples were scored by an experienced gynaecologic pathologist, and the histological classification was used as the reference standard.  **Medium risk (misclassification bias)** | QMSP |
| DeStrooper, 2014 | F3 | HR-HPV positive women enrolled in one arm of PROHTECT study, non-responders (self-sample brush). Those with HR-HPV-positive initial screen invited for colposcopy. | Samples obtained from population based randomised controlled screening POBASCAM trial.  As for C11  **Medium risk (misclassification bias)** |  |
| Huang, 2010 | PS6 |  | Women referred for low or high grade cytology (cases) underwent colposcopy directed cervical biopsy. Health women attending routine cervical screening acted as controls who did not undergo biopsy.  **High risk (misclassification bias)** |  |
| **Studies among women with HPV16 infection** | | | | |
| Louvanto, 2016 | E3 | Women with abnormal pap referred for colposcopy and matched with women presenting for annual cytology (Pap test) matched by age and site; irrespective of HPV positivity. | Women referred to colposcopy due to abnormal cytology, all with biopsy taken (cases) and women attending routine cervical screening (controls; unclear if these women had biopsy taken).  **Medium risk (misclassification bias)** | PSQ |
| Bryant, 2016 | H16-1 | Women aged 20-22 attending first call for cervical screening as part of a cervical screening programme; included all women who screened positive for HPV16 and were referred for colposcopy (n=267), a further 22 who tested positive for HPV16 and with normal cytology. | Women were referred for colposcopy if abnormal cytology. Patients referred for colposcopy were managed according to National Health Service UK guidelines.  Unclear biopsy indication and histology verification.  **High risk (misclassification bias)** | PSQ |
| Mirabello, 2015 | H16-2 | Exfoliated cervical cell specimens from 99 women, previously reported to contain HPV16 DNA, were randomly selected for a nested case-control study that consisted of 59 precancer cases and 40 controls. Controls were defined as women with HPV16 DNA single infections at enrolment with less than CIN2 histology (if a biopsy was obtained) and cytology that was benign, ASCUS or LSIL during the follow-up study period. The cases were defined as women with HPV16 DNA, single infection at enrolment, with cervical precancer, CIN2–3, at enrolment. | Women with HPV16 single infection with cervical precancer, CIN2-3 acted as cases; Women with HPV16 single infection with <CIN2 histology (if biopsy was obtained) and cytology ≤LSIL acted as controls.  Unclear if all women had biopsy/histology endpoints.  **Medium-high risk** | PSQ & NGS |
| Qiu, 2015 | H16-3 | Patients undergoing routine LBC test at Third Affiliated Hospital of Zhengzhou University; women with abnormal cytology referred for cervical biopsy; Selected women were HPV16 positive. | All women had cervical cytology. Women with abnormal colposcopy had cervical biopsy (unclear if all women were referred for colposcopy).  Three pathologists, blinded to HPV results, established the final cervical biopsies diagnosis.  **High risk (misclassification bias)** | qPCR/MS-HRM (me-sensitive high-resolution melting |
| Simanaviciene, 2015 | H16-4 | Samples from a prior study estimating prevalence of HR-HPV among Lithuanian women with cervical pathology. Selected women were HPV16-positive. | No information on conditions for colposcopy referral or biopsy indication, but all women included in the analysis had histological endpoints.  **Medium risk** | Bisulfite sequencing |
| Brandsma, 2014 | H16-5 | Two populations enrolled 21 women from routine cervical cancer screening program in New Haven and 12 women in Dakar never before screened attending a community health clinic for reasons unrelated to cervical cancer. All women were HPV-16positive. | No information on conditions for colposcopy referral or biopsy indication but all women included in the analysis had histological endpoints. Biopsies from women recruited in USA were collected within 3 months of the cytology sample, same day biopsies were obtained from women in Senegal.  **Medium risk** | Bisulfite sequencing |
| Lorincz, 2013 | H16-7 | All HPV16 positive samples from clinical trial among women with newly diagnosed low grade (borderline changes or mild dysplasia) cytological abnormalities; all women invited for colposcopy at 6 months with biopsy. | Women referred for colposcopy if cytology ASCUS+, colposcopy directed biopsy according to Cervical Screening Wales programme. All women included in the analysis had histology endpoint.  **Medium risk** | PSQ |
| Mirabello, 2013 | H16-8 | Samples from population based cohort of 10,049 individuals recruited for screening and followed up as part of natural history study of HPV among women >18 years. Women referred to colposcopy if cytology abnormal. This study includes HPV16 positive women; 100 with infection clearance in >2 yrs., 38 with persistence withou CIN2+; 67 with HR-HPV persistence and CIN2+. | Women referred to colposcopy if abnormal cytology, abnormal visual inspection or by cervicography (unclear biopsy indication). All women included in this analysis had histology endpoint.  **Medium risk** | PSQ |
| Kottaridi, 2017 | H16-9 | Prospective study including nonpregnant women, 21–67 years of age who attended the gynaecology clinics (May 2013 to 2015) from 3 University Hospitals (Ioannina/Athens, Greece and Imperial College London, United Kingdom. All cervical samples that tested positive for HPV16 and had available histology (punch biopsy or conisation–gold standard) were included. If histology was available from both biopsies and cones, the most severe lesion was used. | No information on conditions for colposcopy referral or biopsy indication, but all women included in the analysis had histological endpoints.  **Medium risk** | PSQ |

ASCUS=Atypical squamous cells of undetermined significance ; CIN=cervical intraepithelial neoplasia; HR-HPV= high-risk human papillomavirus; IQR=interquartile range; LBP=liquid -based cytology ; LSIL=low-grade squamous intraepithelial lesion ; NR=not reported; POU4F3=pou Class 4 Homeobox 3; PSQ=pyrosequencing; qMSP=quantitative methylation-specific polymerase chain reaction; RCT=randomised controlled trial; VIA=visual inspection with acetic acid

**Supplementary Table 3. CpG sites targeted for each gene marker, by study**

| **Author, year** | **Study ID** | **Country** | **Genes studied** | **CpG sites targeted** | **Primer sequences (5' → 3')** |
| --- | --- | --- | --- | --- | --- |
| VanBaars 2016^[45]^ | C4 | Spain-Barcelona | *CADM1/MAL* |  | NR (Commercial assay) - PreCursor-M, Self-screen B.V., Amsterdam |
| Van Zummern 2017^[32]^ | C2 | South Africa-Pretoria | *CADM1/MAL/miR124-2* |  | NR (Commercial assay) - PreCursor-M, Self-screen B.V., Amsterdam |
| DeStrooper 2014^[46]^ | C7 | The Netherlands | *CADM1/MAL/MIR124-2* |  | NR (Commercial assay) - PreCursor-M, Self-screen B.V., Amsterdam |
| Kim 2016^[44]^ | C3 | South Korea-Seoul | *CADM1* | NR | F: TTGTTTTGTTAATTAGGGGATTTG; R (biotin): CACACCCAATACATCTAACCTA; S: GGTGTAAGGTGAGTGA |
| Verhoef 2015^[23]^ | C6 | The Netherlands | *CADM1* | M12, M18 | M12 [F: GCGTCGTCGAACGTTAGCGT; R: AACCAATCACAACGCCCGCG; 6–FAM 50–30 TAMRA probe: CCTCCCCCACCCCGCCCCCT]; M18 [F: ATTTTATTAGTTGTTGGTTCGGGT; R: CTCGACAACACTACTCGCC; 6–FAM 50–30 TAMRA probe: ACCTACCTCAAACTAACGACGTTAACTACCTCCGA] |
| DeStrooper 2014^[47]^ | C8 | The Netherlands | *CADM1* | M12, M18 | M12 [F: GCGTCGTCGAACGTTAGCGT; R: AACCAATCACAACGCCCGCG; 6–FAM 50–30 TAMRA probe: CCTCCCCCACCCCGCCCCCT]; M18 [F: ATTTTATTAGTTGTTGGTTCGGGT; R: CTCGACAACACTACTCGCC; 6–FAM 50–30 TAMRA probe: ACCTACCTCAAACTAACGACGTTAACTACCTCCGA] |
| Hesselink 2011^[48]^ | C11 | The Netherlands | *CADM1* | M12, M18 | M12 [F: GCGTCGTCGAACGTTAGCGT; R: AACCAATCACAACGCCCGCG; 6–FAM 50–30 TAMRA probe: CCTCCCCCACCCCGCCCCCT]; M18 [F: ATTTTATTAGTTGTTGGTTCGGGT; R: CTCGACAACACTACTCGCC; 6–FAM 50–30 TAMRA probe: ACCTACCTCAAACTAACGACGTTAACTACCTCCGA] |
| Overmeer 2011 ^[49]^ | C12 | The Netherlands | *CADM1* | M12, M18 | M12 [F: GCGTCGTCGAACGTTAGCGT; R: AACCAATCACAACGCCCGCG; 6–FAM 50–30 TAMRA probe: CCTCCCCCACCCCGCCCCCT]; M18 [F: ATTTTATTAGTTGTTGGTTCGGGT; R: CTCGACAACACTACTCGCC; 6–FAM 50–30 TAMRA probe: ACCTACCTCAAACTAACGACGTTAACTACCTCCGA] |
| Hesselink 2014^[30]^ | C10 | The Netherlands | *CADM1* | M18 | F: ATTTTATTAGTTGTTGGTTCGGGT; R: CTCGACAACACTACTCGCC; 6–FAM 50–30 TAMRA probe: ACCTACCTCAAACTAACGACGTTAACTACCTCCGA |
| DeVuyst 2015^[33]^ | C5 | Kenya-Nairobi | *CADM1* | M18 | F: ATTTTATTAGTTGTTGGTTCGGGT; R: CTCGACAACACTACTCGCC; 6–FAM 50–30 TAMRA probe: ACCTACCTCAAACTAACGACGTTAACTACCTCCGA |
| Kim 2016^[44]^ | C3 | South Korea-Seoul | *MAL* | NR | F: AAGGTGAGTGAAGGAAATTTGTAA ; R (biotin): CCAAAAAACCAATCTAACTTCTTA; S: TAAAAAAATCCTCTATCCC |
| Verhoef 2015^[23]^ | C6 | The Netherlands | *MAL* | M1, M2 | M1 [F: GCGTAGTATTAAGTAGAGAGGTTCG R: AATAAAAAATAAAACCGACCGC; 6–FAM 50–30 TAMRA probe: ACTAAACCGACGCTAATTCGACGACGCT]; M2 [F: TTAGGTTATTGGGTTTCGCG; R: GTACTAACGTCGACCTTAAAACGA; 6–FAM 50–30 TAMRA probe: TCCGCGCAAACCTCTCGCTAAC] |
| DeStrooper 2014^[47]^ | C8 | The Netherlands | *MAL* | M1, M2 | M1 [F: GCGTAGTATTAAGTAGAGAGGTTCG R: AATAAAAAATAAAACCGACCGC; 6–FAM 50–30 TAMRA probe: ACTAAACCGACGCTAATTCGACGACGCT]; M2 [F: TTAGGTTATTGGGTTTCGCG; R: GTACTAACGTCGACCTTAAAACGA; 6–FAM 50–30 TAMRA probe: TCCGCGCAAACCTCTCGCTAAC] |
| Verhoef 2014^[24]^ | C9 | The Netherlands | *MAL* | M1, M2 | M1 [F: GCGTAGTATTAAGTAGAGAGGTTCG R: AATAAAAAATAAAACCGACCGC; 6–FAM 50–30 TAMRA probe: ACTAAACCGACGCTAATTCGACGACGCT]; M2 [F: TTAGGTTATTGGGTTTCGCG; R: GTACTAACGTCGACCTTAAAACGA; 6–FAM 50–30 TAMRA probe: TCCGCGCAAACCTCTCGCTAAC] |
| Hesselink 2011^[48]^ | C11 | The Netherlands | *MAL* | M1, M2 | M1 [F: GCGTAGTATTAAGTAGAGAGGTTCG R: AATAAAAAATAAAACCGACCGC; 6–FAM 50–30 TAMRA probe: ACTAAACCGACGCTAATTCGACGACGCT]; M2 [F: TTAGGTTATTGGGTTTCGCG; R: GTACTAACGTCGACCTTAAAACGA; 6–FAM 50–30 TAMRA probe: TCCGCGCAAACCTCTCGCTAAC] |
| Overmeer 2011 ^[49]^ | C12 | The Netherlands | *MAL* | M1, M2 | M1 [F: GCGTAGTATTAAGTAGAGAGGTTCG R: AATAAAAAATAAAACCGACCGC; 6–FAM 50–30 TAMRA probe: ACTAAACCGACGCTAATTCGACGACGCT]; M2 [F: TTAGGTTATTGGGTTTCGCG; R: GTACTAACGTCGACCTTAAAACGA; 6–FAM 50–30 TAMRA probe: TCCGCGCAAACCTCTCGCTAAC] |
| DeVuyst 2015^[33]^ | C5 | Kenya-Nairobi | *MAL* | M1 | F: GCGTAGTATTAAGTAGAGAGGTTCG R: AATAAAAAATAAAACCGACCGC; 6–FAM 50–30 TAMRA probe: ACTAAACCGACGCTAATTCGACGACGCT |
| Hesselink 2014^[30]^ | C10 | The Netherlands | *MAL* | M1 | F: GCGTAGTATTAAGTAGAGAGGTTCG R: AATAAAAAATAAAACCGACCGC; 6–FAM 50–30 TAMRA probe: ACTAAACCGACGCTAATTCGACGACGCT |
| DeVuyst 2015^[33]^ | C5 | Kenya-Nairobi | *hsa-miR-124-2* |  | F: GGGTAATTAATTTGGATTTACGTCGTTAT; R: CGTAAAAATATAAACGATACGTATACCTACGT; P: TTTACAACACACGCCTAAA -Xsprobe |
| Verhoef 2014^[24]^ | C9 | The Netherlands | *hsa-miR-124-2* |  | F: GGGTAATTAATTTGGATTTACGTCGTTAT; R: CGTAAAAATATAAACGATACGTATACCTACGT; P: TTTACAACACACGCCTAAA -Xsprobe |
| Hesselink 2014^[30]^ | C10 | The Netherlands | *hsa-miR-124-2* |  | F: GGGTAATTAATTTGGATTTACGTCGTTAT; R: CGTAAAAATATAAACGATACGTATACCTACGT; P: TTTACAACACACGCCTAAA -Xsprobe |
| DeStrooper 2016^[27]^ | F1 | The Netherlands | *hsa-miR-124-2* |  | F: GGGTAATTAATTTGGATTTACGTCGTTAT; R: CGTAAAAATATAAACGATACGTATACCTACGT; P: TTTACAACACACGCCTAAA -Xsprobe |
| Luttmer 2016^[28]^ | F2 | The Netherlands | *FAM194A* | NR | NR |
| DeStrooper 2014^[64]^ | F3 | The Netherlands | FAM194A | NR | NR |
| DeStrooper 2016^[27]^ | F1 | The Netherlands | *FAM19A4* | NR | NR |
| Bu, 2018*^[35]^ | F4 | China-Guangdong | FAM194A | NR | F: 5’-CGGGCGGTTCGGTTAATT-3’ ; R: 5’-AAAACGACGCGCAACTAAC-3′ |
| Kocsis 2017^[29]^ | P1 | Hungary | *POU4F3* | CONFIDENCE Marker^TM^ | NR |
| Kelly 2018 (BF)^[39]^ | E1 | Burkina Faso | *EPB41L3* | 438, 427, 425 | F: GGGGGATTTGTGTAAATTGG; R: (B)- ACCTAAAAACCTCCCTAAAATC; S=GGGATTTGTGTAAATTGG |
| Kelly 2018 (SA) ^[39]^ | E1 | South Africa | *EPB41L3* | 438, 427, 425 | F: GGGGGATTTGTGTAAATTGG; R: (B)- ACCTAAAAACCTCCCTAAAATC; S=GGGATTTGTGTAAATTGG |
| Louvanto 2015^[56]^ | E3 | Canada-Montreal | *EPB41L3* | 438, 427, 425 | F: GGGGGATTTGTGTAAATTGG; R: (B)- ACCTAAAAACCTCCCTAAAATC; S=GGGATTTGTGTAAATTGG |
| Lorincz 2016^[40]^ | E2 | UK-London | *EPB41L3* | 376 to 458 | F: GGGGGATTTGTGTAAATTGG; R: (B)- ACCTAAAAACCTCCCTAAAATC; S: GGGATTTGTGTAAATTGG |
| Vasiljevic 2014^[25]^ | E4 | UK-London | *EPB41L3* | 376 to 458 | F: GGGGGATTTGTGTAAATTGG; R: (B)- ACCTAAAAACCTCCCTAAAATC; S: GGGATTTGTGTAAATTGG |
| Cook, 2018^[10]^ | E8 | Canada-British Colombia | *EPB41L3* | 376 to 458 | F: GGGGGATTTGTGTAAATTGG; R: (B)- ACCTAAAAACCTCCCTAAAATC; S: GGGATTTGTGTAAATTGG |
| Brentnall 2014^[31]^ | H16-6 | UK-London | *EPB41L3* | 376 to 458 | F: GGGGGATTTGTGTAAATTGG; R: (B)- ACCTAAAAACCTCCCTAAAATC; S: GGGATTTGTGTAAATTGG |
| Boers 2014^[50]^ | E5 | The Netherlands | *EPB41L3* | 19071, 19072 | F: GGGATAGTGGGGTTGACGC; R: ATAAAAATCCCGACGAACGA |
| Eijinsk 2012^[51]^ | E6 | The Netherlands-Groningen | *EPB41L3* | 19071, 19072 | F: GGGATAGTGGGGTTGACGC; R: ATAAAAATCCCGACGAACGA |
| van Leeuwan, 2018^[26]^ | E7 | Slovenia | *EPB41L3* | 19071, 19072 | F: GGGATAGTGGGGTTGACGC; R: ATAAAAATCCCGACGAACGA |
| Boers, 2016^[52]^ | E9 | The Netherlands-Groningen | *EPB41L3* | 19071, 19072 | F: GGGATAGTGGGGTTGACGC; R: ATAAAAATCCCGACGAACGA |
| Rogeri, 2018^[34]^ | E11 | Brazil-Sao Paulo | *EPB41L3* | 5543706 - 5543818 | F: GGGATAGTGGGGTTGACGC; R: ATAAAAATCCCGACGAACGA |
| Lin 2011^[38]^ | PS5 | Taiwan | *PAX1* | "M", "U" | "M" [F: TATTTTGGGTTTGGGGTCGC; R: CCCGAAAACCGAAAACCG]; "U" [F: GTTTATTTTGGGTTTGGGGTTGTG; R: CACCCAAAAACCAAAAACCAC] |
| Kim 2016^[44]^ | C3 | South Korea-Seoul | *PAX1* | NR | F: GGGGAGTAGTGAAGGGAATTAATGA; R (biotin): CCCAAACCCAAAATAAACTTCAT; S: AGTGAAGGGAATTAATGAGT |
| Xu 2015^[41]^ | PS1 | China-Shanghai | *PAX1* | NR | F:5’-AAGTTTATTTTGGGTTTGGGGT-3’, outside reverse: 5’-ACCCACCTCATCAACCCTCCC-3’; inside forward, 5’-GTGGAGAGTGTTTTGGGAGGG-3’, in-side reverse, 5’-AAATAACCRAAACTAAACCC-3’ |
| Li 2015^[37]^ | PS4 | China-Weifang | *PAX1* | NR | F: 5’-CGGGAATTAATGAGTTGTTAATT-3’; R: 5’-AAACCCAAAATAAACTTCATCC-3’ |
| Huang 2010^[53]^ | PS6 | Taiwan | *PAX1* | NR | F: 5'CGGTTAGACGAATTTTTTTTAATCGGATGA'3; R: 5'CCCGCGACCCCAAAC'3; Taqman probe: FAM-5'CGCCCGCTCCAAAACCTA'3-TAMARA)  sites. The |
| Kan 2014^[36]^ | PS2 | Taiwan-Taipei | *PAX1* | NR | NR |
| Lai 2014^[42]^ | PS3 | Taiwan | *PAX1, SOX1* | NR | NR |
| Tian, 2017^[43]^ | PS8 | Taiwan | *PAX1, SOX1* | NR | NR |
| van Leeuwan, 2018^[26]^ | E7 | Slovenia | *SOX1* | 27153, 27159 | F: TTGTAGTTTTCGAGTTGGAGGTC; R: AAAACGATACGCTAAACCCG |
| Rogeri, 2018^[34]^ | E11 | Brazil-Sao Paulo | *SOX1* | 112720889 -112721027 | F: GTTATGTAAATTATCGTCGTCGTC; R: AACCCGAAATAAACTACCCG |
| Lorincz 2016^[40]^ | E2 | UK-London | *HPV16 L1* | 6367, 6389 | F: (B)TATATTAAAATGGTGTTAGAATTATATGG; R: ACCCCAACAAATACCATTATTATAA; s: CTAACAAACATTTATTCCCTTC |
| Louvanto 2015^[56]^ | E3 | Canada-Montreal | HPV16 L1 | 6367, 6389 | F: (B)TATATTAAAATGGTGTTAGAATTATATGG; R: ACCCCAACAAATACCATTATTATAA; s: CTAACAAACATTTATTCCCTTC |
| Cook, 2018^[10]^ | E8 | Canada-British Colombia | *HPV16 L1* | 6367, 6389 | F: (B)TATATTAAAATGGTGTTAGAATTATATGG; R: ACCCCAACAAATACCATTATTATAA; s: CTAACAAACATTTATTCCCTTC |
| Brentnall 2014^[31]^ | H16-6 | UK-London | *HPV16 L1* | 6367, 6389 | F: (B)TATATTAAAATGGTGTTAGAATTATATGG; R: ACCCCAACAAATACCATTATTATAA; s: CTAACAAACATTTATTCCCTTC |
| Mirabello 2013^[59]^ | H16-8 | Costa-Rica-Guanacaste | HPV16 L1 | 6367, 6389 | F: (B)TATATTAAAATGGTGTTAGAATTATATGG; R: ACCCCAACAAATACCATTATTATAA; s: CTAACAAACATTTATTCCCTTC |
| Lorincz 2013^[54]^ | H16-7 | UK-Wales | HPV16-L1 | 6367, 6389 | F: (B)TATATTAAAATGGTGTTAGAATTATATGG; R: ACCCCAACAAATACCATTATTATAA; s: CTAACAAACATTTATTCCCTTC |
| Brandsma 2014^[62]^ | H16-5 | Senegal-Dakar/USA-New Haven | HPV16-L1 | 6365, 6387, 6579 | F: TTT AGA TTA TAT TAA AAT GGT GTT AGA ATT; R: CAA ATA ATT AAT TAC CCC AAC AAA TAC |
| Qiu 2015^[60]^ | H16-3 | China-Zhengzhou | HPV16-L1 | 5555 - 5659 | F: GCGCATTATTGTTGATG-TAGGTGATTTTTATTTATATTTTAG, R: GCCGCACTAAA-CAACCAAAAAAACATCTAAAAAAAAATA |
| Kottaridi, 2017^[55]^ | H16-9 | Greece-Athens & UK-London | HPV16 L1 | 5611, 5726, 5927, 6367, 6389, 6457, 6581, 6650, 6796, 7091, 7136, 7145 | NR |
| Mirabello 2015^[58]^ | H16-2 | USA-California | HPV16-L1 | 5602, 5608, 5611, 5617 | F: biotin-TAATAATTTTTTTTGGTGGTGTAT; R: CACTAAACAACCAAAAAAACATCT |
| Brandsma 2014^[62]^ | H16-5 | Senegal-Dakar/USA-New Haven | HPV16-L1 | 5724, 5925 | R: AAA CAC TAA CAT TTT CTA TAT CAT CCA ATT |
| Mirabello 2015^[58]^ | H16-2 | USA-California | HPV16-L1 | 7136, 7145 | F: TTTGTAGATTTAGATTAGTTTTTTTTAGGA; R: biotin- ACTAATTCAACATACATACAATACTTACAA |
| Simanaviciene 2015^[61]^ | H16-4 | Lithuania-Vilnius | HPV16-L1 | 7136, 7145 | Unclear |
| Bryant 2015^[57]^ | H16-1 | Uk-Cardiff | HPV16-L1/L2 | 5600, 5606, 5609, 5615 | Sense: biotin-TTATTGTTGATGTAGGTGATTT; antisense: CCCAATAACCTCACTAAACAACC; sequencing: TAACCTCACTAAACAACCAA |
| Lorincz 2016^[40]^ | E2 | UK-London | *HPV16 L2* | 4238, 4247, 4259, 4268, 4275; | F: GTATGTTTTATAAAGTTGGGTAG; R(B): btn-TTAATAAACTATTATCACTTAACAATAC |
| Cook, 2018^[10]^ | E8 | Canada-British Colombia | *HPV16 L2* | 4238, 4247, 4259, 4268, 4275; | F: GTATGTTTTATAAAGTTGGGTAG; R(B): btn-TTAATAAACTATTATCACTTAACAATAC |
| Brentnall 2014^[31]^ | H16-6 | UK-London | *HPV16 L2* | 4238, 4247, 4259, 4268, 4275; | F: GTATGTTTTATAAAGTTGGGTAG; R(B): btn-TTAATAAACTATTATCACTTAACAATAC |
| Brandsma 2014^[62]^ | H16-5 | Senegal-Dakar/USA-New Haven | HPV16-L2 | 5126, 5171 | F: GTT TTA GAT TTT GAT TTT TTG GAT ATA GTT; R: CCT TAA CAC CTA TAA ATT TTC CAC TAC |
| Lorincz 2013^[54]^ | H16-7 | UK-Wales | HPV16-L2 | 4238, 4247, 4259, 4268, 4275; | F: GTATGTTTTATAAAGTTGGGTAG; R(B): btn-TTAATAAACTATTATCACTTAACAATAC |
| Brandsma 2014^[62]^ | H16-5 | Senegal-Dakar/USA-New Haven | HPV16-E5 | 3887, 3937, 3941 | F: ATA GGA TAT AAT GTA AAA TAT AAA AGT GTA; R: ATA TAT AAA CAC AAA CAA AAA CAA C |
| Lorincz 2016^[40]^ | E2 | UK-London | *HPV18 L2* | 4256, 4261, 4265, 4269, 4275, 4282 | F: GTATAGGTTGTTTTATATAGTGTATTGT; R=(B1)-TCCACCTTAAAAACAACATCAAATAA; S=TGTATTTTTGTAATAAAAGTATGGTA |
| Cook, 2018^[10]^ | E8 | Canada-British Colombia | *HPV18 L2* | 4256, 4261, 4265, 4269, 4275, 4282 | F: GTATAGGTTGTTTTATATAGTGTATTGT; R=(B1)-TCCACCTTAAAAACAACATCAAATAA; S=TGTATTTTTGTAATAAAAGTATGGTA |
| Brentnall 2014^[31]^ | H16-6 | UK-London | *HPV18 L2* | 4256, 4261, 4265, 4269, 4275, 4282 | F: GTATAGGTTGTTTTATATAGTGTATTGT; R=(B1)-TCCACCTTAAAAACAACATCAAATAA; S=TGTATTTTTGTAATAAAAGTATGGTA |
| Lorincz 2016^[40]^ | E2 | UK-London | *HPV31 L1* | 6352, 6364 | F: (B)- ATTTGTGTATTTGAAGTAATTATGGAG; R=TCCAAATTATCTTAAAATAATTACTAAACC; S1=AAATAATTACTAAACCATATAAC; S2=CATTTTTTTAATAAATCAAACAC; S3=CCTACTAACTTATATATTAAAAACT; S4=ACATACTTTCCTACACCT |
| Cook, 2018^[10]^ | E8 | Canada-British Colombia | *HPV31 L1* | 6352, 6364 | F: (B)- ATTTGTGTATTTGAAGTAATTATGGAG; R=TCCAAATTATCTTAAAATAATTACTAAACC; S1=AAATAATTACTAAACCATATAAC; S2=CATTTTTTTAATAAATCAAACAC; S3=CCTACTAACTTATATATTAAAAACT; S4=ACATACTTTCCTACACCT |
| Brentnall 2014^[31]^ | H16-6 | UK-London | *HPV31 L1* | 6352, 6364 | F: (B)- ATTTGTGTATTTGAAGTAATTATGGAG; R=TCCAAATTATCTTAAAATAATTACTAAACC; S1=AAATAATTACTAAACCATATAAC; S2=CATTTTTTTAATAAATCAAACAC; S3=CCTACTAACTTATATATTAAAAACT; S4=ACATACTTTCCTACACCT |
| Lorincz 2016^[40]^ | E2 | UK-London | *HPV33 L2* | 5557, 5560, 5566, 5572 | F: AGGTAGGTATATTGTGGTTTTATTAGGT; R(B): btn-CACATCTAACCCATTTATTCCTATTTC |
| Cook, 2018^[10]^ | E8 | Canada-British Colombia | *HPV33 L2* | 5557, 5560, 5566, 5572 | F: AGGTAGGTATATTGTGGTTTTATTAGGT; R(B): btn-CACATCTAACCCATTTATTCCTATTTC |

F: forward primer; R: reverse primer; P: probe; Xsprobe: minor groove binder probe

**Supplementary Table 4. Pooled (crude) Odds Ratio for the association of methylation with CIN2, CIN3 and invasive cervical cancer (ICC) compared to those with ≤CIN1**

|  | **N studies** | **N women** | **Pooled OR^1^ (95% CI)** | **I^2^** | **p-value** |
| --- | --- | --- | --- | --- | --- |
| ***CIN2 vs. ≤CIN1*** |  |  |  |  |  |
| **All genes combined^2^** | **25** | **5208** | **2.83 (2.01-4.00)** | **63.0%** | **<0.001** |
| *CADM1* | 2 | 308 | 2.79 (1.48-5.25) | 0.0% | 0.566 |
| *MAL* | 2 | 308 | 2.27 (0.73-7.09) | 52.7% | 0.146 |
| *MIR-124-2* | 1 | 248 | 3.17 (1.64-6.13) | - | - |
| *FAM19A4* | 2 | 726 | 2.12 (1.37-3.27) | 0.0% | 0.352 |
| *POU4F3* | 1 | 1287 | 9.87 (2.74-35.60) | - | - |
| *EPB41L3* | 6 | 1211 | 2.35 (1.35-4.08) | 52.2% | 0.063 |
| *PAX1* | 5 | 1372 | 3.78 (0.68-20.88) | 86.5% | <0.001 |
| *SOX1* | 3 | 754 | 1.61 (0.50-5.15) | 68.6% | 0.041 |
| HPV16L1/L2 viral genes | 3 | 304 | 7.45 (2.80-19.80) | 0.0% | 0.691 |
| ***CIN3 vs. ≤CIN1*** |  |  |  |  |  |
| **All genes combined^3^** | **27** | **5378** | **7.92 (6.10-10.29)** | **43.2%** | **0.010** |
| *CADM1* | 3 | 478 | 6.33 (3.35-11.96) | 0.0% | 0.849 |
| *MAL* | 3 | 478 | 2.13 (0.53-8.58) | 68.8% | 0.041 |
| *MIR-124-2* | 1 | 248 | 6.68 (3.11-14.38) | - | - |
| *FAM19A4* | 2 | 726 | 7.81 (4.93-12.39) | 0.0% | 0.593 |
| *POU4F3* | 1 | 1287 | 23.21 (11.04-48.80) | - | - |
| *EPB41L3* | 6 | 1211 | 7.70 (4.77-12.44) | 30.4% | 0.207 |
| *PAX1* | 5 | 1372 | 11.45 (4.86-26.98) | 62.5% | 0.031 |
| *SOX1* | 3 | 754 | 7.64 (4.12-14.19) | 33.9% | 0.220 |
| HPV16L1/L2 viral genes | 3 | 304 | 16.41 (5.41-49.80) | 0.0% | 0.869 |
| ***ICC vs. ≤CIN1*** |  |  |  |  |  |
| **All genes combined^4^** | **15** | **3311** | **32.11 (22.51-45.79)** | **0.0%** | **0.982** |
| *CADM1* | 1 | 170 | 25.72 (7.11-93.13) | - | - |
| *MAL* | 2 | 230 | 24.44 (9.45-63.24) | 0.0% | 0.584 |
| *FAM19A4* | 2 | 726 | 74.69 (0.86-6474.80) | 0.0% | 0.963 |
| *EPB41L3* | 2 | 486 | 36.78 (18.62-72.67) | 0.0% | 0.597 |
| *PAX1* | 4 | 909 | 39.29 (20.54-75.17) | 0.0% | 0.453 |
| *SOX1* | 2 | 519 | 29.19 (11.72-72.68) | 0.0% | 0.627 |
| HPV16L1/L2 viral genes | 2 | 271 | 17.57 (3.89-79.33) | 0.0% | 0.819 |
| ***CIN3 vs. CIN2*** |  |  |  |  |  |
| **All genes combined^5^** | **27** | **5452** | **2.95 (2.03-4.27)** | **70.8%** | **<0.001** |
| *CADM1* | 2 | 308 | 2.57 (1.12-5.89) | 0.0% | 0.454 |
| *MAL* | 2 | 308 | 1.68 (0.78-3.63) | 0.0% | 0.657 |
| *MIR-124-2* | 1 | 248 | 2.11 (0.87-5.11) | - | - |
| *FAM19A4* | 2 | 726 | 3.55 (1.76-7.14) | 22.0% | 0.257 |
| *POU4F3* | 1 | 1287 | 2.35 (0.54-10.25) | - | - |
| *EPB41L3* | 7 | 1455 | 2.76 (1.31-5.84) | 82.0% | <0.001 |
| *PAX1* | 5 | 1372 | 4.64 (2.26-9.51) | 0.0% | 0.828 |
| *SOX1* | 3 | 754 | 4.14 (2.03-8.44) | 0.0% | 0.837 |
| HPV16L1/L2 viral genes | 4 | 514 | 2.75 (1.05-7.17) | 0.0% | 0.914 |
|  |  |  |  |  |  |

^1^all Odds Ratio (OR) are unadjusted for confounders; nineteen studies provided data for DNA methylation positivity for individual human genes or HPV16 L1/L2 viral genes by CIN grade (*Analysis 1*) which included three for *CADM1*^[3-5]^; three for *MAL*^[3-5]^; one for *MIR*^[5]^; two for *FAM19A4*^[6, 7]^; one for *POU4F3*^[8]^; seven for *EPB41L3*^[9-14]^; five for *PAX1*^[3, 15-18]^; three for *SOX1*^[13, 15, 18]^ and four for HPV16^[12, 19-21]^; ^2\^Nine methylation markers from 16 studies representing 24 discrete meta-analysis points (Study C5 studied 3 genes, C4 studied 2 genes, C12 studies 3 genes, PS3 studied 2 genes, E1 studied same gene in two countries); ^3^Nine methylation markers from 17 studies representing 26 discrete meta-analysis points (Study C3 studied 3 genes, C5 studied 3 genes, C4 studied 2 genes, C12 studies 3 genes, PS3 studied, E1 studied same gene in two countries); ^4^Six methylation markers from 7 studies representing 10 discrete meta-analysis points (Study C3 studied 3 genes, PS3 studied 2 genes); ^5^Seven methylation markers from 13 studies representing 19 discrete meta-analysis points (Study C5 studied 3 genes, C4 studied 2 genes, PS3 studied 2 genes, Study E3 studied 2 genes; E1 studied same gene in two countries); refer to Supplementary Figures 2-5 for forest plots;

**Supplementary Table 5. Meta analysis of the performance of individual DNA methylation assays for detection of CIN2+ and CIN3+ ***

|  | **DNA methylation**  **marker** | **N studies** | **N women** | **Pooled Sensitivity**  **(95%CI)** | ***I2*** | **Pooled Specificity**  **(95% CI)** | ***I2*** |
| --- | --- | --- | --- | --- | --- | --- | --- |
| ***All studies, irrespective of threshold level*** |  |  |  |  |  |  |  |
| **CIN2+ vs. ≤CIN1** | *CADM1/MAL/MIR-124-2 ^a^ ^[3-5, 22-27]^* | 9 | 2 772 | 55.3 (39.9-69.8) | *90.8 (86.2-95.4)* | 72.6 (61.7-81.3) | *90.5 (85.6-95.3)* |
|  | *FAM19A4^[6, 7, 28, 29]^* | 4 | 1 134 | 60.0 (55.0-65.0) | *0.0 (0.0-100.0)* | 73.0 (70.0-77.0) | *11.9 (0.0-100.0)* |
|  | *EPB41L3 ^b [10-14, 30-33]^* | 10 | 3 548 | 60.2 (57.3-63.1) | *0.0 (0.0-97.4)* | 74.6 (69.6-79.0) | *79.3 (67.0-91.6)* |
|  | *PAX1^[17, 34-36]^* | 5 | 1 255 | 55.8 (30.7-78.2) | *90.3 (83.3-97.2)* | 88.5 (79.3-93.9) | *93.5 (89.4-97.6)* |
|  | HPV16 L1/L2^c[19-21, 37-42]^ | 9 | 2 556 | 73.5 (57.3-85.2) | *95.6 (93.8-97.4)* | 72.8 (65.9-78.7) | *55.8 (22.8-88.8)* |
| **CIN3+ vs. ≤CIN2** | *CADM1/MAL/MIR124-2 ^a [5, 22-27, 43, 44]^* | 10 | 3 025 | 71.3 (63.2-78.2) | *73.8 (57.4-90.3)* | 70.5 (65.2-75.3) | *87.6 (81.2-94.0)* |
|  | *EPB41L3 ^b [9-11, 13, 14, 31, 33]^* | 8 | 1 674 | 75.3 (66.0-82.6) | *73.6 (56.9-90.3)* | 71.9 (67.8-75.6) | *84.5 (76.0-93.0)* |
|  | *PAX1^[15, 17, 18, 35, 36]^* | 5 | 1 217 | 68.4 (53.0-80.6) | *78.1 (59.0-97.3)* | 83.9 (74.2-90.4) | *90.9 (84.6-97.3)* |
|  | HPV16 L1/L2^c [19, 20, 37, 42]^ | 4 | 504 | 58.8 (34.7-79.3) | *92.6 (86.9-98.2)* | 80.0 (59.4-91.6) | *83.5 (68.2-98.9)* |
| ***Set threshold to achieve 70% specificity*** |  |  |  |  |  |  |  |
| **CIN2+ vs. ≤CIN1** | *CADM1/MAL/MIR-124-2^a [4, 5, 24, 26, 27]^* | 5 | 1 925 | 58.5 (48.2-68.2) | *81.4 (65.7-97.1)* | 69.8 (67.0-72.4) | *0.0 (0.0-100.0)* |
|  | *EPB41L3 ^b [10, 12, 13, 31-33]^* | 7 | 2 775 | 61.0 (58.0-64.0) | *0.0 (0.0-100.0)* | 70.0 (68.0-72.0) | *0.0 (0.0-100.0)* |
|  | HPV16 L1/L2^c [20, 37-42]^ | 7 | 2 316 | 76.8.0 (66.5-84.6) | *87.9 (80.3-95.4)* | 70.4 (67.7-72.9) | *0.0 (0.0-100.0)* |
| **CIN3+ vs. ≤CIN2** | *CADM1/MAL/MIR-124-2^a[4, 5, 24, 26, 43]^* | 6 | 2 230 | 66.0 (58.0-73.0) | *60.5 (25.1-95.9)* | 70.0 (67.0-72.0) | *0.0 (0.0-100.0)* |
|  | *EPB41L3 ^b [9, 10, 13, 14, 31, 33]^* | 7 | 1 494 | 76.0 (66.0-84.0) | *65.6 (37.9-93.4)* | 70.0 (67.0-72.0) | *0.0 (0.0-100.0)* |

*only presenting studies with sufficient data to run meta-analysis; ^a^CADM1, MAL or MIR in any combination ; ^b^includes 8 studies evaluating *EPB41L3* alone and 3 studies evaluating *EPB41L3* as part of the S5 classifier; ^c^ evaluated among women positive for HPV16 DNA; includes 1 study evaluated S5 classifier among women with HPV16 infection^[40]^.

**Supplementary Table 6. Pooled Positive Predictive Value (PPV) of DNA methylation assays for detection of CIN2+ and CIN3+, according to study design**

|  | **Model 1^1^** | |  | **Model 2^2^** | | | | | | |
| --- | --- | --- | --- | --- | --- | --- | --- | --- | --- | --- |
| **CIN2+ detection** | **Observed Prevalence** | **PPV (95%CI)** | ***p-het*** | **Estimated Prevalence** | | | | | | |
|  |  |  |  | **5%** | | **10%** | **20%** | **30%** | **40%** | **50%** |
|  |  |  |  | **PPV (95%CI)** | | **PPV (95%CI)** | **PPV (95%CI)** | **PPV (95%CI)** | **PPV (95%CI)** | **PPV (95%CI)** |
| All studies (n=38 studies) | 36.7% | 57.6 (50.3-64.6) | *25.1 (17.1-35.2)* | 12.1 (10.2-14.0) | | 22.6 (19.4-25.7) | 39.6 (35.3-43.9) | 52.9 (48.4-57.4) | 63.6 (59.4-67.8) | 72.4 (68.8-76.0) |
| Referral population based (n=8) | 27.2% | 43.4 (32.5-55.1) | *23.4 (9.7-46.4)* | 10.7 (9.4-12.0) | | 20.2 (18.0-22.4) | 36.3 (33.1-39.5) | 49.4 (46.0-52.8) | 60.3 (57.0-63.6) | 69.5 (66.6-72.4) |
| Cohort (n=7) | 24.3% | 45.0 (33.2-57.4) | *23.8 (8.7-50.7)* | 13.3 (9.1-17.5) | | 24.5 (17.7-31.2) | 42.2 (33.3- 51.1) | 55.6 (46.5-64.6) | 66.1 (57.9-74.2) | 74.5 (67.5-81.4) |
| Case-Control (n=5) | 33.0% | 51.5 (36.4 to 66.4) | *15.7 (4.5-42.3)* | 11.6 (7.5-15.6) | | 21.6 (14.9-28.3) | 38.3 (29.0-47.6) | 51.6 (41.7-61.4) | 62.3 (53.1-71.6) | 71.3 (63.2-79.4) |
| Convenience (n=9) | 36.4% | 59.6 (46.8-71.3) | *12.6 (4.8-29.1)* | 5.8 (1.4-10.2) | 11.5 (3.3-19.8) | | 22.7 (8.5-36.9) | 33.5 (15.5-51.5) | 43.9 (24.1-63.8) | 54.0 (34.0-74.1) |
| HPV16 positive (n=9) | 58.3% | 79.0 (71.4-85.0) | *22.8 (9.5-45.2)* | 12.5 (9.4-15.6) | 23.2 (18.1-28.2) | | 40.4 (33.6-47.2) | 53.7 (46.7-60.8) | 64.4 (57.9-70.9) | 73.1 (67.5-78.6) |
|  |  |  |  |  | |  |  |  |  |  |
|  | **Model 1^1^** | |  | **Model 2^2^** | | | | | | |
| **CIN3+ detection** | **Observed Prevalence** | **PPV (95%CI)** | ***p-het*** | **Estimated Prevalence** | | | | | | |
|  |  |  |  | **5%** | | **10%** | **20%** | **30%** | **40%** | **50%** |
|  |  |  |  | **PPV (95%CI)** | | **PPV (95%CI)** | **PPV (95%CI)** | **PPV (95%CI)** | **PPV (95%CI)** | **PPV (95%CI)** |
| All studies (n=30 studies) | 21.5% | 40.8 (33.9-48.0) | *17.0 (10.2-27.0)* | 12.7 (11.0-14.5) | | 23.6 (20.8-26.3 | 41.0 (37.2-44.7) | 54.3 (50.5-58.1) | 64.9 (61.4-68.4) | 73.5 (70.5-76.5) |
| Referral population based (n=6) | 17.4% | 31.9 (23.3-41.9) | *5.9 (1.5-20.4)* | 10.6 (9.5-11.8) | | 20.1 (18.1-22.0) | 36.1 (33.3-38.9) | 49.2 (46.2-52.2) | 60.1 (57.2-63.0) | 69.3 (66.8-71.9) |
| Cohort (n=5) | 17.7% | 39.8 (30.8-49.5) | *6.3 (1.2-27.2)* | 14.9 (9.3-20.4) | | 26.9 (18.2-35.6) | 45.3 (34.4-56.3) | 58.7 (48.0-71.69.4) | 68.8 (59.4-78.3) | 76.8 (68.9-84.7) |
| Case-Control (n=5) | 14.8% | 28.0 (20.1-37.5) | *14.4 (5.4-32.1)* | 11.1 (9.6-12.6) | | 20.9 (18.3-23.4) | 37.2 (33.6-40.9) | 50.4 (46.5-54.3) | 61.3 (57.6-65.0) | 70.3 (67.1-73.6) |
| Convenience (n=10) | 21.5% | 45.5 (34.2-57.2) | *5.1 (1.5-16.2)* | 13.9 (11.3-16.6) | 25.5 (21.3-29.7) | | 43.5 (38.1-48.9) | 56.9 (51.5-62.3) | 67.2 (62.4-72.1) | 75.5 (71.4-79.5) |
| HPV16 positive (n=4) | 43.1% | 67.3 (43.4 to 84.7) | *11.2 (2.2-41.3)* | 13.4 (5.5-21.3) | | 24.6 (12.0-37.2) | 42.4 (25.8-59.0) | 55.8 (39.1-72.5) | 66.2 (51.1-81.4) | 74.6 (61.8-87.5) |

^1^ Model 1: PPV bivariate model from the observed data; ^2^ Model 2: PPV obtained from pooled specifity and sensitivity by study design at different levels of prevalence of disease, PPV=Prev*SE/(Prev*SE+(1-Prev)*(1-Spec)) ; p-het=pvalue for hetergeneity

Supplementary Figure 1. DNA methylation positivity by CIN grade in 19 studies

Pooled methylation positivity stratified by gene marker among 19 studies; HPV16 data restricted to women with HPV16 infection.

**Supplementary Figure 2. Meta-analysis of performance of DNA methylation assays (sensitivity and specificity) for CIN2+ detection in 38 studies**

**Supplementary Figure 3. Meta-analysis of performance of DNA methylation assays (sensitivity and specificity) for CIN3+ detection in 30 studies**

**Supplementary Figure 4. Sensitivity of DNA methylation assays for CIN2+ detection, at set specificity of 70% (panel A), and at 50% (panel B)**

1. (B)

**Supplementary Figure 5. Sensitivity of DNA methylation assays for CIN3+ detection, at set specificity of 70% (panel A), and at 50% (panel B)**

1. (B)

1. Poljak M, Ostrbenk A, Seme K, Ucakar V, Hillemanns P, Bokal EV, et al. Comparison of clinical and analytical performance of the Abbott Realtime High Risk HPV test to the performance of hybrid capture 2 in population-based cervical cancer screening J Clin Microbiol. 2011;49(5):1721-9.

2. Cuzick J, Cadman L, Mesher D, Austin J, Ashdown-Barr L, Ho L, et al. Comparing the performance of six human papillomavirus tests in a screening population Br J Cancer. 2013;108(4):908-13.

3. Kim MK, Lee IH, Lee KH, Lee YK, So KA, Hong SR, et al. DNA methylation in human papillomavirus-infected cervical cells is elevated in high-grade squamous intraepithelial lesions and cancer J Gynecol Oncol. 2016;27(2):e14.

4. van Baars R, van der Marel J, Snijders PJ, Rodriquez-Manfredi A, ter Harmsel B, van den Munckhof HA, et al. CADM1 and MAL methylation status in cervical scrapes is representative of the most severe underlying lesion in women with multiple cervical biopsies Int J Cancer. 2016;138(2):463-71.

5. De Vuyst H, Franceschi S, Plummer M, Mugo NR, Sakr SR, Meijer CJ, et al. Methylation Levels of CADM1, MAL, and MIR124-2 in Cervical Scrapes for Triage of HIV-Infected, High-Risk HPV-Positive Women in Kenya J Acquir Immune Defic Syndr. 2015;70(3):311-8.

6. Luttmer R, De Strooper LM, Berkhof J, Snijders PJ, Dijkstra MG, Uijterwaal MH, et al. Comparing the performance of FAM19A4 methylation analysis, cytology and HPV16/18 genotyping for the detection of cervical (pre)cancer in high-risk HPV-positive women of a gynecologic outpatient population (COMETH study) Int J Cancer. 2016;138(4):992-1002.

7. De Strooper LM, Meijer CJ, Berkhof J, Hesselink AT, Snijders PJ, Steenbergen RD, et al. Methylation analysis of the FAM19A4 gene in cervical scrapes is highly efficient in detecting cervical carcinomas and advanced CIN2/3 lesions Cancer Prev Res (Phila). 2014;7(12):1251-7.

8. Kocsis A, Takacs T, Jeney C, Schaff Z, Koiss R, Jaray B, et al. Performance of a new HPV and biomarker assay in the management of hrHPV positive women: Subanalysis of the ongoing multicenter TRACE clinical trial (n > 6,000) to evaluate POU4F3 methylation as a potential biomarker of cervical precancer and cancer Int J Cancer. 2017;140(5):1119-33.

9. Boers A, Bosgraaf RP, van Leeuwen RW, Schuuring E, Heideman DA, Massuger LF, et al. DNA methylation analysis in self-sampled brush material as a triage test in hrHPV-positive women Br J Cancer. 2014;111(6):1095-101.

10. Kelly HA, Chikandiwa A, Warman R, Segondy M, Sawadogo B, Vasiljevic N, et al. Associations of human gene EPB41L3 DNA methylation and cervical intraepithelial neoplasia in women living with HIV-1 in Africa Aids. 2018;32(15):2227-36.

11. Eijsink JJ, Lendvai A, Deregowski V, Klip HG, Verpooten G, Dehaspe L, et al. A four-gene methylation marker panel as triage test in high-risk human papillomavirus positive patients Int J Cancer. 2012;130(8):1861-9.

12. Louvanto K, Franco EL, Ramanakumar AV, Vasiljevic N, Scibior-Bentkowska D, Koushik A, et al. Methylation of viral and host genes and severity of cervical lesions associated with human papillomavirus type 16 Int J Cancer. 2015;136(6):E638-45.

13. van Leeuwen RW, Ostrbenk A, Poljak M, van der Zee AGJ, Schuuring E, Wisman GBA. DNA methylation markers as a triage test for identification of cervical lesions in a high risk human papillomavirus positive screening cohort Int J Cancer. 2018.

14. Boers A, Wang R, van Leeuwen RW, Klip HG, de Bock GH, Hollema H, et al. Discovery of new methylation markers to improve screening for cervical intraepithelial neoplasia grade 2/3 Clin Epigenetics. 2016;8:29.

15. Lai HC, Ou YC, Chen TC, Huang HJ, Cheng YM, Chen CH, et al. PAX1/SOX1 DNA methylation and cervical neoplasia detection: a Taiwanese Gynecologic Oncology Group (TGOG) study Cancer Med. 2014;3(4):1062-74.

16. Li SR, Wang ZM, Wang YH, Wang XB, Zhao JQ, Xue HB, et al. Value of PAX1 Methylation Analysis by MS-HRM in the Triage of Atypical Squamous Cells of Undetermined Significance Asian Pac J Cancer Prev. 2015;16(14):5843-6.

17. Lin CJ, Lai HC, Wang KH, Hsiung CA, Liu HW, Ding DC, et al. Testing for methylated PCDH10 or WT1 is superior to the HPV test in detecting severe neoplasms (CIN3 or greater) in the triage of ASC-US smear results Am J Obstet Gynecol. 2011;204(1):21.e1-7.

18. Tian Y, Yuan Wu NY, Liou YL, Yeh CT, Cao L, Kang YN, et al. Utility of gene methylation analysis, cytological examination, and HPV-16/18 genotyping in triage of high-risk human papilloma virus-positive women Oncotarget. 2017;8(37):62274-85.

19. Simanaviciene V, Popendikyte V, Gudleviciene Z, Zvirbliene A. Different DNA methylation pattern of HPV16, HPV18 and HPV51 genomes in asymptomatic HPV infection as compared to cervical neoplasia Virology. 2015;484:227-33.

20. Brandsma JL, Harigopal M, Kiviat NB, Sun Y, Deng Y, Zelterman D, et al. Methylation of twelve CpGs in human papillomavirus type 16 (HPV16) as an informative biomarker for the triage of women positive for HPV16 infection Cancer Prev Res (Phila). 2014;7(5):526-33.

21. Qiu C, Zhi Y, Shen Y, Gong J, Li Y, Li X. High-resolution melting analysis of HPV-16L1 gene methylation: A promising method for prognosing cervical cancer Clin Biochem. 2015;48(13-14):855-9.

22. Hesselink AT, Heideman DA, Steenbergen RD, Coupe VM, Overmeer RM, Rijkaart D, et al. Combined promoter methylation analysis of CADM1 and MAL: an objective triage tool for high-risk human papillomavirus DNA-positive women Clin Cancer Res. 2011;17(8):2459-65.

23. Van Zummeren M, Kremer WW, Van Aardt MC, Breytenbach E, Richter KL, Rozendaal L, et al. Selection of women at risk for cervical cancer in an HIV-infected South African population Aids. 2017;31(14):1945-53.

24. De Strooper LM, Hesselink AT, Berkhof J, Meijer CJ, Snijders PJ, Steenbergen RD, et al. Combined CADM1/MAL methylation and cytology testing for colposcopy triage of high-risk HPV-positive women Cancer Epidemiol Biomarkers Prev. 2014;23(9):1933-7.

25. De Strooper LM, van Zummeren M, Steenbergen RD, Bleeker MC, Hesselink AT, Wisman GB, et al. CADM1, MAL and miR124-2 methylation analysis in cervical scrapes to detect cervical and endometrial cancer J Clin Pathol. 2014;67(12):1067-71.

26. Verhoef VM, Heideman DA, van Kemenade FJ, Rozendaal L, Bosgraaf RP, Hesselink AT, et al. Methylation marker analysis and HPV16/18 genotyping in high-risk HPV positive self-sampled specimens to identify women with high grade CIN or cervical cancer Gynecol Oncol. 2014;135(1):58-63.

27. Verhoef VM, van Kemenade FJ, Rozendaal L, Heideman DA, Bosgraaf RP, Hesselink AT, et al. Follow-up of high-risk HPV positive women by combined cytology and bi-marker CADM1/MAL methylation analysis on cervical scrapes Gynecol Oncol. 2015;137(1):55-9.

28. De Strooper LM, Verhoef VM, Berkhof J, Hesselink AT, de Bruin HM, van Kemenade FJ, et al. Validation of the FAM19A4/mir124-2 DNA methylation test for both lavage- and brush-based self-samples to detect cervical (pre)cancer in HPV-positive women Gynecol Oncol. 2016;141(2):341-7.

29. Bu Q, Wang S, Ma J, Zhou X, Hu G, Deng H, et al. The clinical significance of FAM19A4 methylation in high-risk HPV-positive cervical samples for the detection of cervical (pre)cancer in Chinese women BMC Cancer. 2018;18(1):1182.

30. Rogeri CD, Silveira HCS, Causin RL, Villa LL, Stein MD, de Carvalho AC, et al. Methylation of the hsa-miR-124, SOX1, TERT, and LMX1A genes as biomarkers for precursor lesions in cervical cancer Gynecol Oncol. 2018;150(3):545-51.

31. Lorincz AT, Brentnall AR, Scibior-Bentkowska D, Reuter C, Banwait R, Cadman L, et al. Validation of a DNA methylation HPV triage classifier in a screening sample Int J Cancer. 2016;138(11):2745-51.

32. Vasiljevic N, Scibior-Bentkowska D, Brentnall AR, Cuzick J, Lorincz AT. Credentialing of DNA methylation assays for human genes as diagnostic biomarkers of cervical intraepithelial neoplasia in high-risk HPV positive women Gynecol Oncol. 2014;132(3):709-14.

33. Cook DA, Krajden M, Brentnall AR, Gondara L, Chan T, Law JH, et al. Evaluation of a validated methylation triage signature for human papillomavirus positive women in the HPV FOCAL cervical cancer screening trial Int J Cancer. 2018.

34. Xu J, Xu L, Yang B, Wang L, Lin X, Tu H. Assessing methylation status of PAX1 in cervical scrapings, as a novel diagnostic and predictive biomarker, was closely related to screen cervical cancer Int J Clin Exp Pathol. 2015;8(2):1674-81.

35. Kan YY, Liou YL, Wang HJ, Chen CY, Sung LC, Chang CF, et al. PAX1 methylation as a potential biomarker for cervical cancer screening Int J Gynecol Cancer. 2014;24(5):928-34.

36. Huang TH, Lai HC, Liu HW, Lin CJ, Wang KH, Ding DC, et al. Quantitative analysis of methylation status of the PAX1 gene for detection of cervical cancer Int J Gynecol Cancer. 2010;20(4):513-9.

37. Bryant D, Hibbitts S, Almonte M, Tristram A, Fiander A, Powell N. Human papillomavirus type 16 L1/L2 DNA methylation shows weak association with cervical disease grade in young women J Clin Virol. 2015;66:66-71.

38. Mirabello L, Schiffman M, Ghosh A, Rodriguez AC, Vasiljevic N, Wentzensen N, et al. Elevated methylation of HPV16 DNA is associated with the development of high grade cervical intraepithelial neoplasia Int J Cancer. 2013;132(6):1412-22.

39. Mirabello L, Frimer M, Harari A, McAndrew T, Smith B, Chen Z, et al. HPV16 methyl-haplotypes determined by a novel next-generation sequencing method are associated with cervical precancer Int J Cancer. 2015;136(4):E146-53.

40. Brentnall AR, Vasiljevic N, Scibior-Bentkowska D, Cadman L, Austin J, Szarewski A, et al. A DNA methylation classifier of cervical precancer based on human papillomavirus and human genes Int J Cancer. 2014;135(6):1425-32.

41. Lorincz AT, Brentnall AR, Vasiljevic N, Scibior-Bentkowska D, Castanon A, Fiander A, et al. HPV16 L1 and L2 DNA methylation predicts high-grade cervical intraepithelial neoplasia in women with mildly abnormal cervical cytology Int J Cancer. 2013;133(3):637-44.

42. Kottaridi C, Kyrgiou M, Pouliakis A, Magkana M, Aga E, Spathis A, et al. Quantitative Measurement of L1 Human Papillomavirus Type 16 Methylation for the Prediction of Preinvasive and Invasive Cervical Disease J Infect Dis. 2017;215(5):764-71.

43. Hesselink AT, Heideman DA, Steenbergen RD, Gok M, van Kemenade FJ, Wilting SM, et al. Methylation marker analysis of self-sampled cervico-vaginal lavage specimens to triage high-risk HPV-positive women for colposcopy Int J Cancer. 2014;135(4):880-6.

44. Overmeer RM, Louwers JA, Meijer CJ, van Kemenade FJ, Hesselink AT, Daalmeijer NF, et al. Combined CADM1 and MAL promoter methylation analysis to detect (pre-)malignant cervical lesions in high-risk HPV-positive women Int J Cancer. 2011;129(9):2218-25.
